# Ketogenic diets inhibit mitochondrial biogenesis and induce cardiac fibrosis

**DOI:** 10.1038/s41392-020-00411-4

**Published:** 2021-02-09

**Authors:** Sha Xu, Hui Tao, Wei Cao, Li Cao, Yan Lin, Shi-Min Zhao, Wei Xu, Jing Cao, Jian-Yuan Zhao

**Affiliations:** 1 Zhongshan Hospital of Fudan University, Obstetrics & Gynecology Hospital of Fudan University, State Key Lab of Genetic Engineering, School of Life Sciences, Key Laboratory of Reproduction Regulation of NPFPC, and Institutes of Biomedical Sciences, Fudan University, 200438 Shanghai, China; 2 Collaborative Innovation Center for Biotherapy, West China Hospital, Sichuan University, 610041 Chengdu, China; 3 Department of Cardiothoracic Surgery, Second Hospital of Anhui Medical University, and Cardiovascular Research Center, Anhui Medical University, 230601 Hefei, China; 4 Department of Anatomy and Neuroscience Research Institute, School of Basic Medical Sciences, Zhengzhou University, 450001 Zhengzhou, China

**Keywords:** Biochemistry, Cardiology, Molecular biology

## Abstract

In addition to their use in relieving the symptoms of various diseases, ketogenic diets (KDs) have also been adopted by healthy individuals to prevent being overweight. Herein, we reported that prolonged KD exposure induced cardiac fibrosis. In rats, KD or frequent deep fasting decreased mitochondrial biogenesis, reduced cell respiration, and increased cardiomyocyte apoptosis and cardiac fibrosis. Mechanistically, increased levels of the ketone body β-hydroxybutyrate (β-OHB), an HDAC2 inhibitor, promoted histone acetylation of the *Sirt7* promoter and activated *Sirt7* transcription. This in turn inhibited the transcription of mitochondrial ribosome-encoding genes and mitochondrial biogenesis, leading to cardiomyocyte apoptosis and cardiac fibrosis. Exogenous β-OHB administration mimicked the effects of a KD in rats. Notably, increased β-OHB levels and SIRT7 expression, decreased mitochondrial biogenesis, and increased cardiac fibrosis were detected in human atrial fibrillation heart tissues. Our results highlighted the unknown detrimental effects of KDs and provided insights into strategies for preventing cardiac fibrosis in patients for whom KDs are medically necessary.

## Introduction

The low-carbohydrate, high-fat ketogenic diet (KD) is a remarkably effective treatment for medically intractable epilepsy and has been applied in the clinical setting for over 70 years.^1^ In addition, KDs have been widely applied in the clinical treatment of various diseases, such as diabetes,^2^ cancer,^3,4^ and neurological disorders, including Alzheimer’s disease and Parkinson’s disease.^5^ KDs are also used by healthy individuals, predominantly to promote weight loss.^6^

Consumption of a KD forces the body to use fats rather than carbohydrates to generate energy. Three major forms of ketone bodies, namely acetoacetate (AcAc), β-hydroxybutyrate (β-OHB), and acetone, are generated in the liver during fatty acid oxidation and transported to extrahepatic tissues by the circulatory system. Circulating total ketone body concentrations in healthy adult humans normally exhibit circadian oscillations of ~100–250 μM. However, levels can reach 1–8 mM after KD consumption, prolonged exercise, or deep fasting and can be as high as 25 mM under pathological conditions, such as diabetic ketoacidosis.^7–10^ β-OHB accounts for 70% of ketone bodies and has been suggested to be beneficial because it not only serves as a vital alternative metabolic fuel source in the fed, fasted, and starved states^11^, but also exerts antioxidative,^12^ antiaging,^13^ and anti-inflammatory effects.^14^

Although numerous reports have acknowledged the beneficial effects of β-OHB, its safety has been challenged by certain clinical lines of evidence related to its effects on cardiovascular health. For example, the concentration of β-OHB in heart tissues is significantly higher in patients with atrial fibrillation (AF).^15^ In addition, increased circulating β-OHB is independently associated with major adverse cardiovascular events in patients undergoing hemodialysis.^16^ Moreover, diabetes, which is usually associated with high levels of ketone bodies, constitutes an independent risk factor for cardiovascular diseases, including AF, coronary heart disease, and stroke.^17,18^ The potential detrimental effects of β-OHB have also been implicated in clinical studies, in which KD has been practiced. For example, the occurrence of cardiovascular disease of unknown etiology has frequently been described in those in the KD group in various studies.^19–21^ Moreover, a 25-year follow-up study of a large cohort found that a low-carbohydrate diet was associated with increased mortality,^22^ although it was not clear whether the KD directly increased the incidence of cardiovascular disease, a major factor that affects life expectancy.^23^ Taken together, these findings suggest that KD consumption or β-OHB accumulation may increase the risks of cardiovascular disease, suggesting that long-term consumption of a KD should be carefully considered.

In this study, we examined the effects of a KD and β-OHB accumulation on cardiovascular biology and health in cultured cells, animal models, and clinical samples in order to shed light on the potential negative effects of KDs and to elucidate the underlying mechanisms.

## Results

### Long-term KD exposure resulted in cardiac fibrosis

To survey the potential pathological effects of a KD on cardiac disease, we fed rats either a KD or normal diet and monitored changes in the rat heart (Supplementary Fig. 1a–c). After 16 weeks, besides a decrease in body weight (Supplementary Fig. 1d), fat mass (Supplementary Fig. 1e), and blood pressure (Supplementary Fig. 1f, g) in KD-fed rats, we observed increased heart rates and impaired cardiac function, as evaluated by echocardiography (Table 1). In KD-fed rats, the left ventricular posterior wall thickness (LVPWd) increased significantly compared with normal diet-fed rats, indicating a compensatory increase in cardiomyocytes, used to enhance cardiac contractility (Table 1). If the left heart function was decompensated, the blood accumulated in the pulmonary circulation. The right ventricular anterior wall thickness (RVAW) also increased, indicating increased pulmonary circulation resistance and corresponding thickening of the right ventricular myocardium. Furthermore, the left atrial diameter (LAD), left ventricular dimension in diastole (LVDd), and left ventricular dimension in systole (LVDs) increased in KD-fed rats, indicating that cardiac function was decompensated and entered a state of failure (Table 1). Taken together, the echocardiography results revealed impaired cardiac function in KD-fed rats.

**Table 1:** Echocardiographic data from rats fed a normal diet, ketogenic diet, or caloric restriction diet

	Normal diet	Caloric restriction	Ketogenic diet
Heart rate, beats/min	321.60 ± 11.06	334.60 ± 16.65	363.20 ± 8.47*
LAD, mm	3.93 ± 0.17	3.87 ± 0.19	4.56 ± 0.23**
RAD, mm	4.08 ± 0.18	4.01 ± 0.20	4.24 ± 0.20
LVAWd, mm	1.89 ± 0.06	1.91 ± 0.05	1.96 ± 0.12
LVPWd, mm	1.79 ± 0.06	1.76 ± 0.09	2.08 ± 0.11*
LVDd, mm	7.46 ± 0.06	7.54 ± 0.11	7.83 ± 0.14*
LVDs, mm	5.81 ± 0.12	5.96 ± 0.10	6.64 ± 0.31**
LVFS, %	42.89 ± 2.99	43.99 ± 2.79	40.14 ± 4.55
LV mass, mg	997.60 ± 26.37	988.28 ± 19.97	1021.06 ± 22.12
RVAW, mm	0.54 ± 0.03	0.55 ± 0.04	0.63 ± 0.04**
RVD, mm	3.80 ± 0.07	3.82 ± 0.08	3.87 ± 0.14
LPV (S), cm/s	52.22 ± 1.42	51.88 ± 1.52	52.32 ± 2.46
LPV (D), cm/s	35.24 ± 1.02	35.48 ± 1.23	35.64 ± 2.51

Values are mean ± SEM (*n* = 6)

*LAD* left atrial diameter, *RAD* right atrial diameter, *LVAWd* left ventricular anterior wall thickness, *LVPWd* left ventricular posterior wall thickness, *LVDd* left ventricular dimension in diastole, *LVDs* left ventricular dimension in systole, *LVFS* left ventricular fractional shortening, *RVAW* right ventricular anterior wall thickness, *RVD* right ventricular dimension, *LPV* left pulmonary venous flow, *S* Systolic, *D* diastolic

*p < 0.05, **p < 0.01 versus the normal diet-fed group according to Student’s *t* tests

**Fig. 1:**
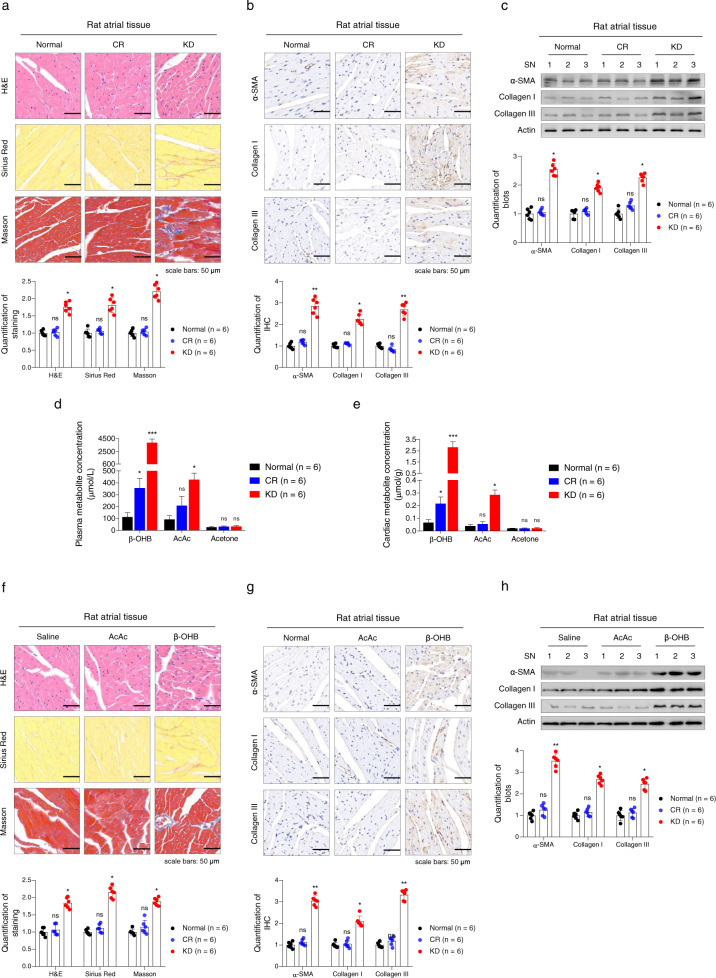
Increased β-OHB induced cardiac fibrosis in animal models. **a**, **b** H&E, Sirius Red, and Masson trichrome staining (**a**) results in atrial tissues from rats fed a normal diet, KD, or CR diet, and immunohistochemical (IHC) analysis of α-SMA, collagen I, and collagen III in atrial tissues from rats according to diet (**b**). Representative results (top) and summary of quantitative analysis are shown. **c** Western blotting analysis of α-SMA, collagen I, and collagen III in atrial tissues of rats fed the different diets. **d**, **e** Ketone body concentrations in the plasma (**d**) and atrial tissues (**e**) of rats fed the different diets. **f**, **g** H&E, Sirius Red, and Masson staining results of atrial tissues from rats intraperitoneally injected with saline, AcAc, or β-OHB (**f**) and IHC analysis of α-SMA, collagen I, and collagen III in atrial tissues from rats according to treatment (**g**). Representative results (top) and summary of quantitative analysis are shown. **h** Western blotting analysis of α-SMA, collagen I, and collagen III in atrial tissues of rats according to treatment. Data shown are means ± standard errors. ^ns^*p* > 0.05, *p < 0.05, **p < 0.01, ***p < 0.001 versus the corresponding control group according to Student’s *t* tests. Some full-length blots are presented in the Supplementary Fig. 13

Considering the fact that increased cardiac fibrosis is associated with cardiac hypertrophy, dilatation,^24–26^ and the onset of AF^27^ and that significantly increased ketone bodies were observed in the cardiac tissues of patients with AF,^15^ we next examined the cardiac fibrosis levels in rat atrial tissues. In accordance with impaired cardiac function, we observed the occurrence of fibrosis in the atrial tissues of KD-fed but not normal diet-fed rats, as analyzed by atrial tissue staining (Fig. 1a and Supplementary Fig. 2a). We also detected the expression levels of protein markers of fibrosis using both immunohistochemistry (IHC) and western blotting. Indeed, type I collagen, type III collagen, and α-smooth muscle actin (α-SMA) levels were increased in atrial tissues from KD-fed rats (Fig. 1b, c and Supplementary Fig. 2b, c). These results indicated that long-term KD feeding may cause cardiac fibrosis.

Because a KD usually provides fewer calories than a carbohydrate-rich diet, we employed caloric restriction (CR) in another group of rats as a control to determine whether KD induced cardiac fibrosis was caused by an insufficient supply of energy. We found CR did not induce cardiac fibrosis and cardiac function impairment in rats (Fig. 1a–c, Supplementary Figs. 1 and 2, and Table 1). Moreover, the KD caused increased levels of β-OHB and AcAc, the predominant forms of ketone bodies, in both plasma and heart tissues (Fig. 1d, e). In contrast, CR only induced a mild elevation of β-OHB and AcAc (Fig. 1d, e). These observations suggested that a considerable elevation in ketone bodies may account for the onset of cardiac fibrosis in rats.

### Increased β-OHB resulted in cardiac fibrosis

To further elucidate whether and which ketone bodies induced fibrosis, we increased the levels of either β-OHB or AcAc in rats by intraperitoneal injection (Supplementary Fig. 3a–d) because β-OHB and AcAc were the predominant forms of ketone bodies elevated after KD feeding (Fig. 1d, e). During the 16-week injection period, water intake, daily calorie intake, body weight, fat mass, and blood pressure were not affected by either β-OHB or AcAc injection (Supplementary Fig. 3e–j). We found that increased levels of β-OHB, but not AcAc, resulted in impaired cardiac function, as evidenced by increased LAD, LVDs, LVDd, LVPWd, and RVAW in echocardiography analysis (Table 2). Furthermore, we observed the occurrence of cardiac fibrosis in β-OHB intraperitoneal injected rats, as evident by atrial staining (Fig. 1f and Supplementary Fig. 4a) and analysis of cardiac fibrosis molecular markers, namely type I collagen, type III collagen, and α-SMA (Fig. 1g, h and Supplementary Fig. 4b, c). We also observed that frequent deep fasting (Supplementary Fig. 5a, b) induced high levels of β-OHB (Supplementary Fig. 5c) and molecular phenotypes of cardiac fibrosis, including characteristic fiber staining results (Supplementary Fig. 5d), and expression of collagen and α-SMA (Supplementary Fig. 5e). Together, these findings supported the theory that increased levels of β-OHB resulted in cardiac fibrosis.

**Table 2:** Echocardiographic data from rats injected with saline, AcAc, or β-OHB

	Saline	AcAc	β-OHB
Heart rate, beats/min	318.80 ± 8.53	324.00 ± 13.75	354.80 ± 15.69*
LAD, mm	3.84 ± 0.23	3.96 ± 0.23	4.50 ± 0.22**
RAD, mm	4.10 ± 0.18	4.11 ± 0.19	4.19 ± 0.21
LVAWd, mm	1.91 ± 0.05	1.92 ± 0.08	2.02 ± 0.12
LVPWd, mm	1.81 ± 0.05	1.79 ± 0.04	2.16 ± 0.24*
LVDd, mm	7.47 ± 0.06	7.58 ± 0.14	7.73 ± 0.18*
LVDs, mm	5.78 ± 0.08	5.87 ± 0.17	6.57 ± 0.53*
LVFS, %	42.69 ± 2.08	44.36 ± 2.52	40.40 ± 4.11
LV mass, mg	1000.20 ± 18.10	1003.60 ± 24.22	1026.00 ± 23.91
RVAW, mm	0.53 ± 0.03	0.56 ± 0.04	0.63 ± 0.03**
RVD, mm	3.82 ± 0.10	3.77 ± 0.08	3.78 ± 0.07
LPV (S), cm/s	52.04 ± 1.25	52.14 ± 1.67	51.62 ± 3.33
LPV (D), cm/s	36.42 ± 1.16	35.72 ± 1.46	35.44 ± 2.24

Values are mean ± SEM (*n* = 6)

*LAD* left atrial diameter, *RAD* right atrial diameter, *LVAWd* left ventricular anterior wall thickness, *LVPWd* left ventricular posterior wall thickness, *LVDd* left ventricular dimension in diastole, *LVDs* left ventricular dimension in systole, *LVFS* left ventricular fractional shortening, *RVAW* right ventricular anterior wall thickness, *RVD* right ventricular dimension, *LPV* left pulmonary venous flow, *S* Systolic, *D* diastolic

*p < 0.05, **p < 0.01 versus the saline group according to Student’s *t* tests

### β-OHB promoted cardiomyocyte apoptosis

**Fig. 2:**
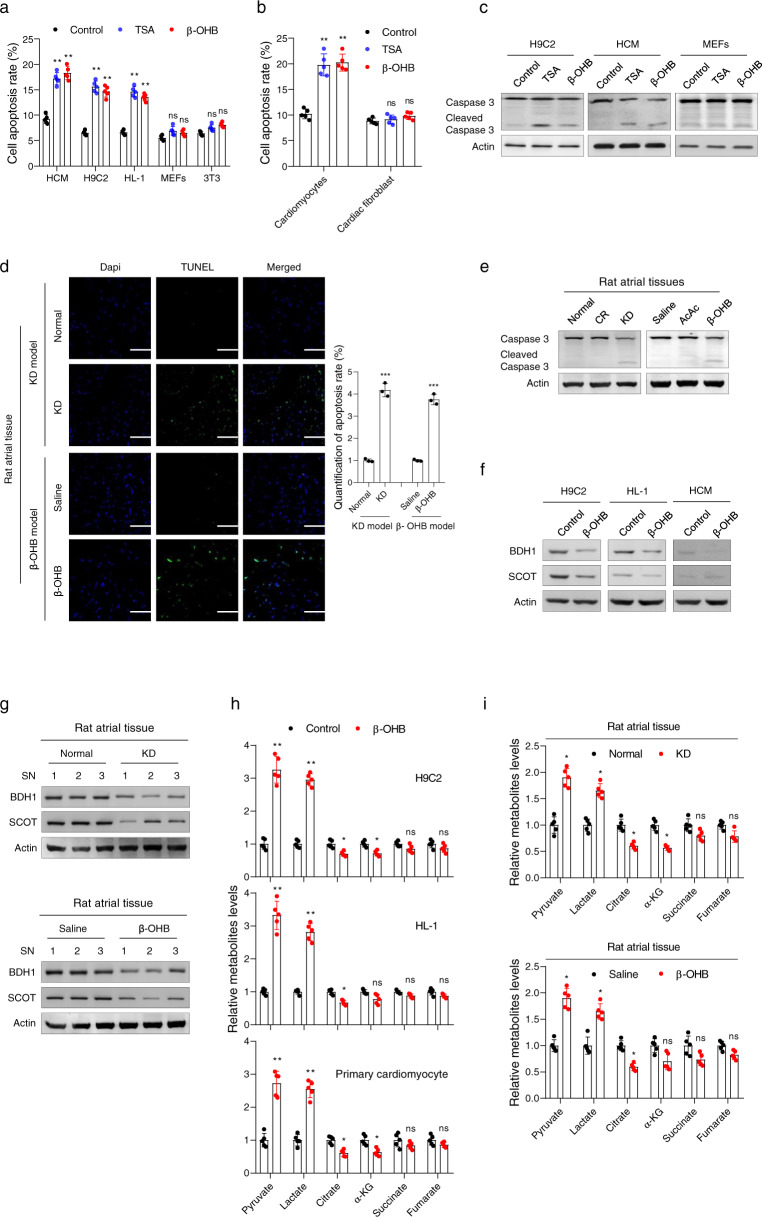
Increased β-OHB promoted cardiomyocyte apoptosis by decreasing mitochondrial metabolism. **a** Cell apoptosis associated with various treatments in different cultured cell lines. Trichostatin A (TSA) is a general histone deacetylase inhibitor; *n* = 5. **b** Cell apoptosis associated with various treatments in primary cells isolated from neonatal mice; *n* = 5. **c** Western blotting analysis of caspase 3 in different cell lines with various treatments. **d** TUNEL analysis of atrial tissues from rats with different treatments; *n* = 3. **e** Western blotting analysis of caspase 3 in atrial tissues from rats with various treatments. **f** Western blotting analysis of BDH1 and SCOT in different cell lines treated with or without β-OHB. **g** Western blotting analysis of BDH1 and SCOT in atrial tissues from rats with various treatments. **h** Metabolite concentrations in cell lines and primary cardiomyocyte treated with or without β-OHB; *n* = 5. **i** Metabolite concentrations in atrial tissues from rats; *n* = 5. Some full-length blots are presented in the Supplementary Fig. 13

We next investigated how β-OHB induced cardiac fibrosis. In cultured human cardiomyocytes (HCM), rat cardiomyoblasts (H9C2), and mouse cardiac muscle cells (HL-1), increased levels of β-OHB led to elevated rates of apoptosis; however, β-OHB did not induce significant increases in apoptosis in fibroblasts, such as mouse embryonic fibroblasts (MEFs) and 3T3 cells (Fig. 2a). In primary cells isolated from neonatal mice, β-OHB treatment also led to an increase in apoptosis in primary mouse cardiomyocytes, but not in primary mouse cardiac fibroblast (Fig. 2b). In accordance with increased apoptosis, elevated β-OHB led to increases in the levels of cleaved caspase 3 in HCM and H9C2 cells, but not in MEFs (Fig. 2c). Moreover, in rat atrial tissue, we confirmed that high β-OHB levels, induced by either KD feeding or β-OHB injection, led to increased rates of apoptosis, as evidenced by in situ TUNEL assays (Fig. 2d) and detection of the levels of cleaved caspase 3 in rat atrial tissue (Fig. 2e). These results suggested that a high concentration of β-OHB caused cell-type-specific cardiomyocyte apoptosis and may underlie β-OHB induced cardiac fibrosis, which is known to be caused by cardiomyocyte apoptosis.^28,29^

We next evaluated the mechanisms underlying cardiomyocyte apoptosis induced by β-OHB. First, given that β-OHB constitutes an important alternative energy source that can be catalyzed into acetyl-CoA for processing through the citrate cycle,^30^ we examined whether β-OHB affected energy metabolism through enhancing the ketolysis pathway. By measuring the expression of proteins within the ketolysis pathway in H9C2, HL-1, and HCM cells treated with or without β-OHB, we observed decreased expression of β-hydroxybutyrate dehydrogenase 1 (BDH1) and succinyl-CoA:3-ketoacid CoA transferase (SCOT) in β-OHB-treated cells (Fig. 2f), and atrial tissues from KD-fed and β-OHB-injected rats (Fig. 2g), suggesting that increased input of ketone bodies did not enhance ketolysis. We next evaluated the glycolytic and citrate metabolic flux in cultured cell lines and primary cardiomyocytes. Decreased levels of citrate cycle metabolites and increased levels of the glycolytic metabolites pyruvate and lactate were observed in β-OHB-treated cells (Fig. 2h). Together with our results showing that decreased levels of citrate cycle metabolites and increased levels of the glycolytic metabolites were also observed in atrial tissues from rats with high β-OHB levels (Fig. 2i), these findings indicated that high β-OHB did not enhance mitochondrial metabolism. Instead, increased β-OHB may lead to reduced mitochondrion numbers because the levels of some mitochondrial enzymes decreased (Fig. 2f, g).

### β-OHB decreased mitochondrion levels in cardiomyocyte

**Fig. 3:**
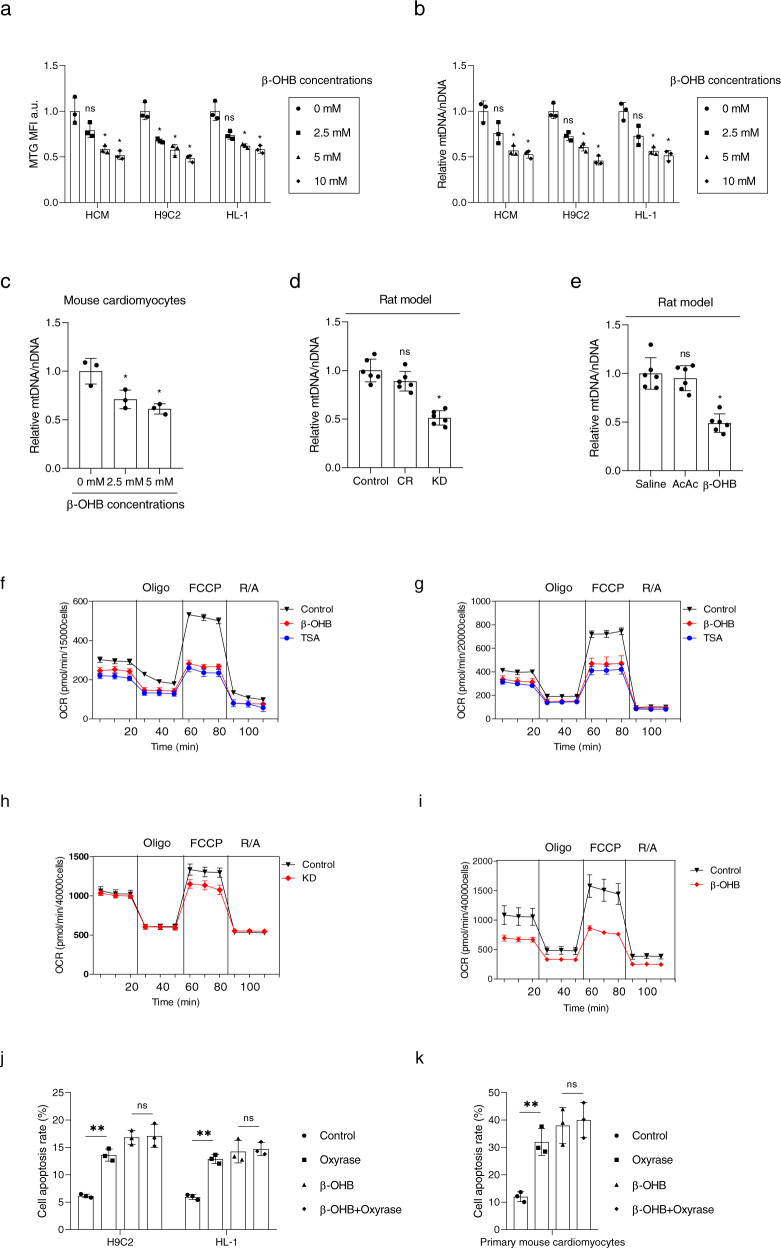
Increased β-OHB decreased mitochondrion levels. **a**, **b** Mitochondrial mass, as determined by MitoTracker Green staining (**a**) and mitochondrial DNA quantification (**b**) in cells with different concentrations of β-OHB; *n* = 3. **c** Mitochondrial mass determined by mitochondrial DNA quantification in mouse primary cardiomyocytes; *n* = 3. **d**, **e** Mitochondrial mass determined by mitochondrial DNA quantification in atrial tissues from rats with different treatments; *n* = 6/group. **f**, **g** Oxygen consumption rates (OCRs) in H9C2 (**f**) and HL-1 (**g**) cells treated with β-OHB or trichostatin A (TSA). **h**, **i** OCRs in primary cardiomyocytes isolated from the atrial tissues of rats with various treatments. **j**, **k** Apoptosis in cell lines and primary mouse cardiomyocytes treated with β-OHB and oxyrase; *n* = 3. Data shown are means ± standard errors. ^ns^*p* > 0.05, *p < 0.05, **p < 0.01, ***p < 0.001 versus the corresponding control group according to Student’s *t* tests

Next, we evaluated the number of mitochondria in response to β-OHB treatment. Our results showed that β-OHB treatment decreased MitoTracker Green staining levels (Fig. 3a) and decreased the ratio of mitochondrial (mt) DNA to nucleic DNA (Fig. 3b) in HCM, H9C2, and HL-1 cells, in a concentration-dependent manner. In primary mouse cardiomyocytes isolated from neonatal mice, β-OHB treatment also led to a decrease in mitochondrion mass, as determined by measuring the ratio of mtDNA to nucleic DNA (Fig. 3c). In accordance with the observations in cultured cells, in rats fed a KD or injected intraperitoneally with β-OHB, significant decreases in cardiac mitochondrion mass were observed compared with those in untreated animals, as determined by measuring the ratio of mtDNA to nucleic DNA (Fig. 3d, e). Moreover, the mtDNA damage levels were not altered significantly in β-OHB-treated cells (Supplementary Fig. 6a) and cardiomyocytes from rats fed a KD or injected intraperitoneally with β-OHB (Supplementary Fig. 6b, c). These results indicated that β-OHB decreased mitochondrial numbers in cultured cells and rat heart tissue.

Consistent with this observation, mitochondrial respiration was significantly reduced after β-OHB treatment in both H9C2 and HL-1 cells (Fig. 3f, g). We also observed decreased mitochondrial respiration in primary cardiomyocytes isolated from KD-fed or β-OHB-injected rats (Fig. 3h, i). Furthermore, oxyrase-induced hypoxia caused apoptosis, and saturated the proapoptotic effects of β-OHB in H9C2 and HL-1 cells (Fig. 3j) and primary mouse cardiomyocytes (Fig. 3k). These results were consistent with the hypothesis that a decrease in mitochondrion numbers may lead to cardiomyocyte apoptosis, which has been shown to be induced by hypoxia.^31–35^ In addition, we observed that β-OHB slightly decreased the mitochondrial mass in MEFs and primary mouse cardiac fibroblasts (Supplementary Fig. 7a, b), and did not exhibit strong inhibitory effects on mitochondrial respiration (Supplementary Fig. 7c, d). Accordingly, the apoptosis levels of MEFs and primary cardiac fibroblasts were not altered in response to hypoxia and β-OHB treatments (Supplementary Fig. 7e, f). These observations suggested that β-OHB caused cell-type-specific cardiomyocyte apoptosis through inhibiting mitochondrial respiration, consistent with previous findings demonstrating that cardiomyocytes are more sensitive to mitochondrial inhibition than fibroblasts.^36–38^ Taken together, these results supported the hypothesis that high levels of β-OHB may induce cardiomyocyte apoptosis by inhibiting mitochondria-mediated oxygen utilization.

### β-OHB impaired mitochondrial biogenesis by activating SIRT7

**Fig. 4:**
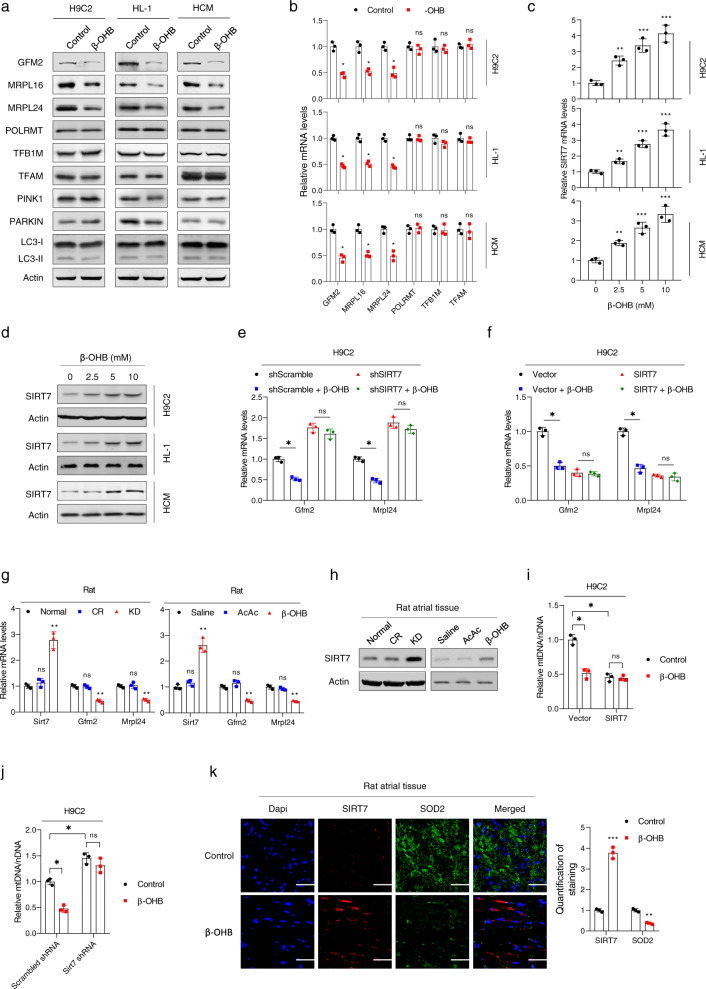
β-OHB impaired mitochondrial biogenesis by activating SIRT7. **a** Western blotting analysis of proteins involved in mitochondrial biogenesis and autophagy in different cell lines treated with or without β-OHB. **b** Analysis of mRNA expression of genes involved in the mitochondrial biogenesis pathway in different cell lines treated with or without β-OHB; *n* = 3. **c** *Sirt7* mRNA levels in different cell lines treated with different concentrations of β-OHB. **d** SIRT7 protein levels in different cell lines treated with different concentrations of β-OHB. **e** *Gfm2* and *Mrpl24* mRNA levels in SIRT7-knockdown H9C2 and control cells treated with or without β-OHB; *n* = 3. **f** *Gfm2* and *Mrpl24* mRNA levels in SIRT7-overexpressing H9C2 cells and control cells treated with or without β-OHB; *n* = 3. **g** *Sirt7*, *Gfm2*, and *Mrpl24* mRNA levels in atrial tissues from rats with different treatments. **h** SIRT7 protein levels in rat atrial tissues according to different treatments. **i**, **j** Mitochondrial mass determined by mitochondrial DNA quantification in H9C2 cells with different treatments; *n* = 3. **k** Double immunofluorescence staining of SIRT7 and SOD2 in rat atrial tissues. Right panel shows the quantitative results of three replicates. Data shown are means ± standard errors. ^ns^*p* > 0.05, *p < 0.05, **p < 0.01, ***p < 0.001 versus the control group according to Student’s *t* tests. Some full-length blots are presented in the Supplementary Fig. 13

By monitoring protein markers of mitochondrial biogenesis and degradation, we found that β-OHB decreased protein levels of mitochondrial ribosomal protein L16 (MRPL16), mitochondrial ribosomal protein L24 (MRPL24), and G elongation factor mitochondrial 2 (GFM2), markers of mitochondrial biogenesis (Fig. 4a).^39^ β-OHB also decreased *GFM2*, *MRPL16*, and *MRPL24* mRNA levels (Fig. 4b). Moreover, the expression of PTEN-induced kinase 1 (PINK1) and parkin (PRKN), along with the relative ratio of LC3-I to LC3-II, markers of mitophagy, were not affected by β-OHB treatment, suggesting that β-OHB did not promote mitochondrial autophagy (Fig. 4a). These results collectively suggested that β-OHB inhibited cell respiration via the suppression of mitochondrial biogenesis.

In an attempt to identify genes that were transcriptionally regulated by β-OHB, we evaluated the gene expression profiles of H9C2 cells with or without β-OHB treatment. The results showed that the transcription of *Sirt4* and *Sirt7* was activated by β-OHB (Supplementary Fig. 8a). Because other markers of mitochondrial biogenesis, such as *Polrmt*, *Tfb1m*, and *Tfam*, were not reduced after β-OHB treatment and because SIRT7 specifically regulates MRPL16, MRPL24, and GFM2,^40^ we hypothesized that β-OHB blocked mitochondrial activity via SIRT7-mediated transcriptional regulation. In cultured H9C2, HL-1, and HCM cells, we confirmed that β-OHB increased SIRT7 expression at both the mRNA and protein levels in a concentration-dependent manner (Fig. 4c, d). Knockdown of SIRT7 increased the transcription of *Gfm2* and *Mrpl24*, and attenuated the inhibitory effects of β-OHB treatment on *Gfm2* and *Mrpl24* transcription (Fig. 4e). Conversely, overexpression of SIRT7 resulted in decreased *Gfm2* and *Mrpl24* transcription, and saturated the effects of β-OHB treatment (Fig. 4f). Moreover, rats with high levels of β-OHB, including KD-fed and β-OHB-injected rats, exhibited increased cardiac SIRT7 mRNA and protein expression (Fig. 4g, h), as well as decreased *Gfm2* and *Mrpl24* mRNA levels (Fig. 4g). Furthermore, overexpression of SIRT7 decreased mitochondrion levels, whereas knockdown of SIRT7 slightly increased mitochondrion levels in cultured cells (Fig. 4i, j). Either overexpressing or knocking down SIRT7 in cells attenuated the inhibitory effects of β-OHB treatment (Fig. 4i, j). Double immunofluorescence staining of atrial tissues from β-OHB-injected or normal rats revealed that β-OHB promoted SIRT7 expression and reduced mitochondrial numbers, as indicated by measuring the level of superoxide dismutase 2 (SOD2); this further indicated that β-OHB impaired mitochondrial biogenesis by activating SIRT7 (Fig. 4k). Furthermore, although high β-OHB induced SIRT4 overexpression, forced SIRT4 overexpression had no effect on *Gfm2* or *Mrpl24* expression (Supplementary Fig. 8b). These results collectively confirmed that β-OHB inhibited mitochondrial biogenesis by promoting SIRT7 overexpression.

### β-OHB inhibited histone deacetylase 2 and activated Sirt7 transcription

**Fig. 5:**
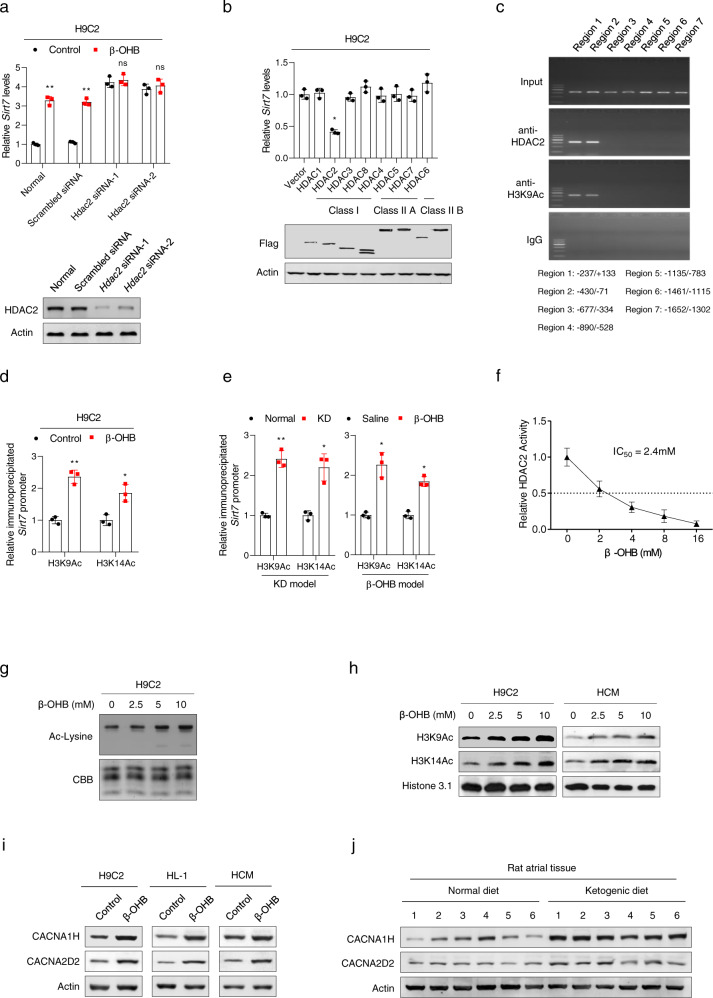
β-OHB activated *Sirt7* transcription by inhibiting HDAC2. **a** *Sirt7* mRNA levels in *Hdac2*-knockdown or control H9C2 cells treated with or without β-OHB; *n* = 3. The lower panel shows the knockdown efficiency. **b** *Sirt7* mRNA levels in H9C2 cells overexpressing different histone deacetylases (HDACs); *n* = 3. The lower panel shows the knockdown efficiency. **c** ChIP followed by PCR showing HDAC2 and H3K9Ac occupancy at the *Sirt7* promoter. **d** ChIP followed by qRT-PCR showing the binding affinities of H3K9Ac and H3K14Ac to the *Sirt7* promoter in cells treated with or without β-OHB; *n* = 3. **e** ChIP followed by qRT-PCR showing the binding affinities of H3K9Ac and H3K14Ac to the *Sirt7* promoter in rats with different treatments; *n* = 3. **f** In vitro assay showing the effects of β-OHB on inhibition of HDAC2 activity (IC_50_: 2.4 mM). **g** Total histone acetylation levels in H9C2 cells treated with different concentrations of β-OHB. **h** H3K9Ac and H3K14Ac levels in H9C2 and HCM cells treated with different concentrations of β-OHB. **i** CACNA1H and CACNA2D2 levels in different cells treated with or without β-OHB. **j** CACNA1H and CACNA2D2 levels in atrial tissues from rats fed a normal diet or KD. Data shown are means ± standard errors. ^ns^*p* > 0.05, *p < 0.05, **p < 0.01, ***p < 0.001 versus the corresponding control group according to Student’s *t* tests. Some full-length blots are presented in the Supplementary Fig. 13

We next investigated how β-OHB regulated the transcription of *Sirt7*. We hypothesized that histone acetylation may play a role, as β-OHB is an established inhibitor of histone deacetylases (HDACs), include HDAC1, HDAC3, HDAC4, and HDAC6.^12^ Knockdown of HDACs individually in β-OHB-treated H9C2 cells revealed that only *Hdac2* knockdown increased *Sirt7* expression and abrogated the ability of β-OHB to activate *Sirt7* transcription (Fig. 5a and Supplementary Fig. 9). In turn, overexpression of HDAC2, but not other HDACs, inhibited *Sirt7* transcription in H9C2 cells (Fig. 5b), indicating that HDAC2 was the HDAC that regulated *Sirt7* transcription. Accordingly, using chromatin immunoprecipitation (ChIP), we found that HDAC2 bound to the core promoter region of *Sirt7* and localized to the same region as acetylated H3K9 (Fig. 5c). Moreover, the acetylation levels of H3K9 and H3K14 in the promoter of *Sirt7* were increased in β-OHB-treated cultured H9C2 cells (Fig. 5d). In addition, we observed increased acetylation levels of H3K9 and H3K14 in the promoter of *Sirt7* in the heart tissues of KD-fed and β-OHB-injected rats, when compared with those in the corresponding control rats (Fig. 5e). These results suggested that *Sirt7* transcriptional activation was related to alterations in acetylation, which were regulated by the β-OHB/HDAC2 pathway.

Although β-OHB was previously shown to be negatively correlated with cellular HDAC2 activity,^41^ direct evidence has not yet been obtained to validate whether β-OHB inhibits HDAC2 deacetylase activity. Therefore, we conducted an in vitro deacetylation assay and found that β-OHB had significant inhibitory effects on HDAC2, with an IC_50_ of 2.4 mM in vitro (Fig. 5f), suggesting that KD-induced elevation of β-OHB in the heart decreased HDAC2 activity by more than half. In cultured cells, we confirmed that β-OHB treatment resulted in increased levels of total histone acetylation (Fig. 5g), particularly of H3K9 and H3K14, downstream targets of HDAC2 (Fig. 5h). Trichostatin A, a general HDAC inhibitor, caused phenotypes similar to those of β-OHB, including activation of *Sirt7* transcription (Supplementary Fig. 10a), downregulation of *Gfm2* and *Mrpl24* expression (Supplementary Fig. 10a), inhibition of mitochondrial biogenesis (Supplementary Fig. 10b), inhibition of mitochondrial respiration (Fig. 3f, g), and promotion of apoptosis in cardiomyocytes (Fig. 2a–c). In addition, we confirmed that the expression of voltage-dependent T-type calcium channel subunit alpha-1H (CACNA1H) and voltage-dependent T-type calcium channel subunit alpha-2 delta-2 (CACNA2D2), which are known to be inhibited by HDAC2,^42^ were increased in β-OHB-treated cells (Fig. 5i) and heart tissues from KD-fed rats (Fig. 5j). Taken together, these results indicated that β-OHB activated *Sirt7* transcription by inhibiting HDAC2.

### Increased β-OHB and its consequences were observed in patients with AF

**Fig. 6:**
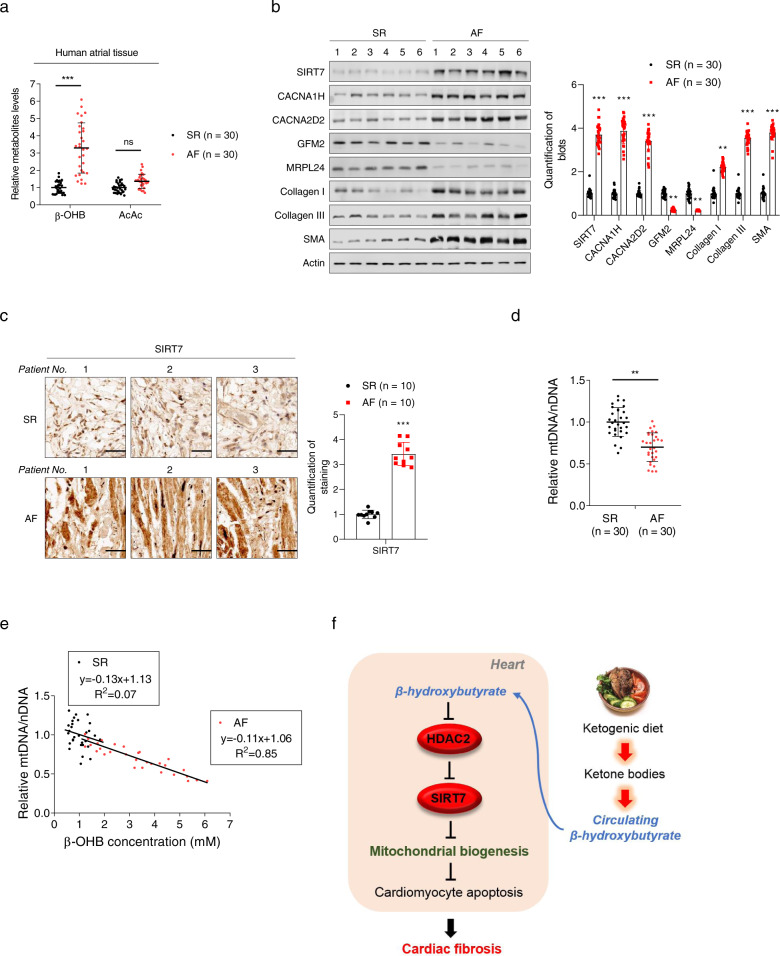
Increased β-OHB was associated with increased risk of atrial fibrillation (AF). **a** Concentrations of β-OHB and AcAc in atrial tissues from patients with sinus rhythm (SR) and AF. **b** The left panel shows representative western blotting analysis of SIRT7, CACNA1H, CACNA2D2, GFM2, MRPL24, collagen I, collagen III, and α-SMA in atrial tissues from patients with SR and AF. The right panel shows the quantitative results. **c** IHC analysis of SIRT7 in atrial tissues from patients with SR and AF. The right panel shows the quantitative results. **d** Mitochondrial mass determined by mtDNA quantification in atrial tissues from patients with SR and AF. **e** Correlation between β-OHB concentration and mitochondrial mass in atrial tissues from patients with SR and AF. **f** Schematic illustration showing how KD increases the risk of cardiac fibrosis. Data shown are means ± standard errors. ^ns^*p* > 0.05, *p < 0.05, **p < 0.01, ***p < 0.001 versus the corresponding control group according to Student’s *t* tests. Some full-length blots are presented in the Supplementary Fig. 13

In order to confirm the causal role of β-OHB in cardiac fibrosis, we examined cardiac fibrosis, β-OHB levels, and changes in β-OHB-associated markers in patients with AF. First, we found that β-OHB levels in cardiac tissues were 3.3-fold higher in patients with AF than in those with sinus rhythm (SR; Fig. 6a). Second, CACNA1H and CACNA2D2 expression was higher in cardiac tissues from patients with AF, indicating that HDAC2 was inhibited in the cardiac tissues of these patients (Fig. 6b and Supplementary Fig. 11a). Third, increased levels of SIRT7 were observed in cardiac tissues from patients with AF compared with those in patients with SR, according to both western blotting (Fig. 6b and Supplementary Fig. 11a) and IHC (Fig. 6c and Supplementary Fig. 11b). Fourth, markers of fibrosis, including type I collagen, type III collagen, and α-SMA, were higher in patients with AF (Fig. 6b and Supplementary Fig. 11a). Lastly, the number of mitochondria was significantly lower in cardiac tissues from patients with AF than in those from patients with SR, as indicated by the ratio of mtDNA to nucleic DNA (Fig. 6d). These findings, together with the observation that the cardiac β-OHB concentration was negatively correlated with the number of mitochondria (Fig. 6e), confirmed that elevations in β-OHB were associated with cardiac fibrosis and an increased risk of AF.

## Discussion

Some studies have indicated that β-OHB exhibits beneficial effects in the cardiac system under pathological conditions and can, for example, be used as an alternative fuel in the human failing heart.^43^ Moreover, this compound improves cardiac cell excitation–contraction coupling during hypoxia^44^ and prevents myocardial damage after coronary occlusion in animal model.^45^ In this study, we provided evidence and mechanistic explanations from cultured cells, animal models, and clinical samples to show that long-term KD-induced β-OHB accumulation was detrimental to heart health by promoting cardiac fibrosis (Fig. 6f).

Several facts support our findings. First, a KD forces cells in the body to rely primarily on fatty acid β-oxidation rather than glycolysis for energy production, inevitably leading to increased ketone body production, primarily in the liver; this change elevates circulating levels of ketone bodies and exposes cardiomyocytes in the heart to high levels of ketone bodies. However, although theoretically all cells in the body are exposed to elevated levels of ketone bodies, cardiomyocytes are among those most vulnerable to high ketone body exposure, as high levels of ketone bodies reduce the mitochondrial content significantly, as demonstrated in our study. The mitochondrial content in cardiomyocytes reaches up to 30% of the total cell volume, much higher than that in the cells of other organs, because the heart requires high levels of energy production from the β-oxidation of fatty acids.^46^ Therefore, although the regulatory effects of β-OHB to mitochondrial content may be present in many organs because of HDAC2 and SIRT7 expression (Supplementary Fig. 12), long-term β-OHB exposure selectively induced apoptosis of cardiomyocytes.

In addition, HDAC2, which was found to be inactivated by β-OHB in our study, is essential for promoting heart development and maintaining heart function,^47–49^ and downregulation of HDAC2 increases apoptosis in cardiomyocytes.^42^ Our in vitro assay showed that β-OHB inhibited HDAC2, with an IC_50_ of 2.4 mM, which is lower than the β-OHB concentrations observed in the human AF heart and in KD-fed and deep-fasted rats (i.e., ~3–4 mM). These results further supported that consumption of a KD induced the accumulation of β-OHB to pathological levels.

Although the β-OHB/HDAC2/SIRT7–mitochondrial biogenesis–cardiac fibrosis pathway was found in the current study, several questions remained unsolved. First, systematic metabolic remodeling induced by KD is more extensive than β-OHB intraperitoneal injection. For example, KD induced decreased levels of glucose and increased levels of free fatty acid in blood, and increased gluconeogenesis in liver and kidney. In contrast, β-OHB intraperitoneal injection did not cause such glucose/fatty acid metabolic reprogram in rat. Although our studies indicated that increased β-OHB was able to cause cardiac fibrosis in rat, whether the dysregulation of other types of metabolites induced by long-term KD contributes to cardiac fibrosis remains unknown. Secondly, in the current study, we did not examine whether long-term KD or β-OHB injection caused other organ abnormalities. So the safety of long-term effects of KD and β-OHB injection still required further studies. Third, besides SIRT7, HDAC2 has many downstream targets. In the current study, we aimed to confirm that the β-OHB/HDAC2/SIRT7 pathway was important to mitochondrial biogenesis. Therefore, we did not measure other cardiac fetal genes that may be regulated by HDAC2. Further studies are required to reveal regulatory effects of HDAC2 on mitochondrial genes. Last, we validated that SIRT7 inhibited mitochondrial biogenesis in cardiomyocytes, consistent with previous findings showing that increased expression or activity of SIRT7 inhibited mitochondrial biogenesis in hematopoietic stem cells and human embryonic kidney cells.^40,50^ However, SIRT7 deficiency in fibroblasts has been shown to induce mitochondrial dysfunction.^51^ Thus, these contradictory observations indicated that SIRT7 may exert different regulatory effects on mitochondrial function in different types of cells.

The findings of our current study defined the mechanism underlying the negative health effects of KDs, which are adopted worldwide for therapeutic purposes and have been increasingly preferred by healthy individuals in order to prevent obesity. Our results strongly suggest that unless the adverse effects of a KD on the cardiac system can be effectively avoided, healthy individuals should reconsider the use of a KD for weight loss.

## Materials and methods

### Animal models

Adult male Sprague-Dawley rats weighing 180–220 g were purchased from the Experimental Animal Center of Anhui Medical University. Detailed descriptions of KD-feeding model, ketone body intraperitoneal injection model, and frequent deep fasting model, are given in the Supplementary Methods online.

### Clinical samples

For western blotting and metabolite quantification, 30 atrial appendages from patients with rheumatic AF (AF group; AF lasting for >6 months), and 30 atrial appendages from patients with SR (SR group) were collected during heart valve replacement surgery or cardiac catheterization conducted between June 2016 and May 2017 at the Department of Cardiothoracic Surgery, The Second Hospital of Anhui Medical University. All patients with AF and SR were matched for sex and age. In the surgery, atrial tissue samples were removed after valve replacement was complete and the incision area was repaired. In cardiac catheterization, left atrial appendage tissue was removed from the heart with a stapler. The removed atrial tissue samples were collected and stored at −80 °C.

### Statistics

Statistical analysis was performed using Prism 6.0 software (GraphPad Software, Inc.), Excel (Microsoft Corp.), and R version 2.17. Results were expressed as means ± standard deviations or standard errors of the means. One-way analysis of variance followed by pairwise comparisons using least significant difference test was performed to test between-groups differences, and two-tailed Student’s *t* test was performed for the two-group analysis. Differences were considered statistically significant if the *p* value was <0.05.

### Study approval

All animal experiments and procedures conformed to the guidelines of Directive 2010/63/EU of the European Parliament on the Protection of Animals Used for Scientific Purposes or the NIH guidelines. All animal experimental procedures were approved by the Animal Care and Use Committee of Anhui Medical University. All human samples experimental procedures were reviewed and approved by the ethics committee of Anhui Medical University. Written informed consent was obtained from all eligible patients, and this study conformed to the guidelines outlined in the Declaration of Helsinki.

### Experimental setup

Detailed descriptions of the experimental setup and chemicals, including echocardiography, histopathology, IHC, cell culture and treatments, cardiac fibroblast and cardiomyocyte isolation and culture, antibodies, plasmid construction and transfection, RNA interference, immunoprecipitation and immunoblotting, ChIP assays, quantitative real-time reverse transcription (qRT)-PCR, in vitro HDAC activity assay, histone extraction, gas chromatography/mass spectrometry, measurements of the concentrations of ketone bodies, apoptosis assay, TUNEL assay, measurements of mitochondrial mass and mtDNA/nDNA, mtDNA damage quantification, measurements of oxygen consumption, and Immunofluorescence, are given in the Supplementary Methods online.

## Supplementary information

Supplementary Materials

Supplementary Methods online

Supplementary Figures and Table

## Data Availability

The data sets used for the current study are available from the corresponding author upon reasonable request.
