# Supplementary material for: Ketogenic diets inhibit mitochondrial biogenesis and induce cardiac fibrosis
Source: Signal Transduct Target Ther. 2021 Feb 9;6:54. doi: 10.1038/s41392-020-00411-4 (PMC7870678; doi:10.1038/s41392-020-00411-4)
Supplement: Supplementary file 2 — Supplementary Methods online [file 41392_2020_411_MOESM2_ESM.docx]

**Supplementary Methods online**

***Animal models***

Adult male Sprague-Dawley rats weighing 180–220 g were purchased from the Experimental Animal Center of Anhui Medical University. In the KD feeding model, three groups of rats (n = 6 rats/group) were fed a normal diet (ad libitum feeding), KD (50 g/kg body mass, ad libitum feeding), or CR diet, in which the animals were given 14 g of chow, constituting 70% of the average daily food intake (approximately 20 g). These three groups of rats were fed the special diets for 4 months. The normal diet contained approximately 9.46% casein, 0.14% L-cystine, 35.1% corn starch, 3.3% maltodextrin 10, 38.27% sucrose, 4.7% cellulose, 2.4% soybean oil, 1.9% cocoa butter, 0.9% mineral mix, 1.2% dicalcium phosphate, 0.5% calcium carbonate, 1.6% potassium citrate, 0.1% vitamin mix, 0.19% choline bitartrate and 0.11% DL-methionine; the KD contained approximately 16.5% casein, 0.25% L-cystine,, 8.2% cellulose, 4.25% soybean oil, 62.7% cocoa butter, 1.6% mineral mix, 2.1% dicalcium phosphate, 0.9% calcium carbonate, 2.7% potassium citrate, 0.16% vitamin mix, 0.32% choline bitartrate and 0.32% DL-methionine (percentages are mass%). Both chows were obtained from Shanghai Nuowei Biotechnology Company (Shanghai, China).

In the ketone body intraperitoneal injection model, we prepared the injection solution and performed injections as follows. (a) In the β-OHB group, β-OHB solution (Sigma-Aldrich, St. Louis, MO, USA, #54965) was prepared in sodium medium. This group of rats (n = 6 rats/group) was intraperitoneally injected with β-OHB at a dosage of 100 mg/kg body mass every other day to induce high levels of β-OHB. (b) In the AcAc group, AcAc was synthetized by base-catalyzed hydrolysis of ethylacetoacetate (ethyl-AcAc, Sigma-Aldrich, #00410). In brief, 3.33 ml of 5 M NaOH was added to 10 ml ethyl-AcAc to make a 5% NaOH solution and heated at 60°C for 30 min. In order to remove the remaining ethanol from the AcAc solution, we continued heating the solution at 70°C for 2 h with stirring. After that, the solution was neutralized with 50% HCl to a pH of 8.0, and the concentration of AcAc was determined with a colorimetric enzyme-based total ketone body assay (Wako, Osaka, Japan) prior to aliquoting and storage at –80°C. This group of rats (n = 6 rats/group) was intraperitoneally injected with AcAc at a dosage of 50 mg/kg body mass every other day to induce high levels of AcAc. (c) In the saline group, to ensure an equal concentration of sodium was used in the different groups, the same volume of normal saline was injected as a control. The concentration of Na^+^ in each group was calculated, and extra was added as necessary to ensure that each group had the same concentration of Na^+^. All injections were performed at 8 am. After 72 h of monitoring of the concentrations of β-OHB and AcAc, we confirmed that performing injections every other day was sufficient for maintaining plasma β-OHB or AcAc at levels 3-fold higher than those in control animals (Supplementary Fig. 3B, C). These three groups of rats were fed a normal diet for 16 weeks.

In the frequent deep fasting model, six rats were allowed to feed freely every other day, while the control group was fed normally; this feeding regimen was followed for 4 months.

After the rats completed the feeding program, all rats were anesthetized and sacrificed, and their hearts and plasma were isolated quickly. Portions of the atrium were fixed in 4% phosphate-buffered paraformaldehyde and prepared for histopathology, or snap-frozen in liquid nitrogen and stored at −80°C for RNA and protein analysis.

***Echocardiography***

Echocardiography was performed with the animals anesthetized by 2% isoflurane. Echocardiography was used to assess left ventricle (LV) systolic and diastolic function using a 2-dimensional guided M-mode ultrasound system (Acuson Sequoia, Siemens Medical Solutions, Mountain View, California). Two-dimensional images from a short-axis view of the LV at the level of the papillary muscles were obtained using a 15 MHz linear transducer at a paper speed of 100 mm/s. The echocardiography was performed before opening the chest (baseline) and during the time course of the experiment in all groups. Three consecutive cardiac cycles were analyzed, and the average was used for data analysis.

***Blood pressure measurement***

The rat blood pressure was measured by tail-cuff plethysmography (CODA Monitor, Kent) in non-anesthetized animals. Briefly, the resting rat was fixed on a plastic restrainer at room temperature. The proximal tail was attached to the computer via a 4-channel dynamic signal acquisition system. The average of six consecutive measurements was taken as the mean blood pressure of each animal.

***Histopathology***

Cardiac tissue specimens were fixed in 4% neutral buffered formalin and then embedded in paraffin for light microscopy examination. Tissue sections (4 μm thick) were stained with hematoxylin and eosin (HE), Sirius Red, and Masson trichrome stain. HE staining was used to evaluate acidophilic and basic tissue components in cardiac sections. The cross-sectional sizes of cardiomyocytes were analyzed with AxioVision software. For Sirius Red staining, fibrosis was visualized using a Picrosirius Red staining kit (Polysciences Inc.). Quantification of the fibrotic area was performed with Image-Pro Plus (Media Cybernetics). Representative left ventricle cross-sections were stained with Picrosirius Red. For Masson trichrome staining, fibrosis was visualized using a Masson’s trichrome staining kit. For analysis of the collagen volume fraction (CVF), the collagen area and total area in images were quantified with Image-Pro Plus, and assessment of CVF was performed using the following formula: CVF = collagen area / total area.

***Immunohistochemistry***

Tissues were fixed in 4% neutral buffered formalin and then embedded in paraffin. Formalin-fixed tissues were deparafﬁnized and rehydrated. After high-pressure cooking retrieval and blocking with 5% bovine serum albumin (BSA), sections were incubated overnight at 4°C with one of the following primary antibodies: anti-collagen I (1:150), anti-collagen III (1:150), or anti-α-SMA (1:100). Primary antibodies were detected with rabbit anti-mouse and goat anti-rabbit non-biotinylated regents. Quantitative evaluations were performed with a Photo and Image Autoanalysis System (Image-Pro-Plus).

***Measurements of the concentrations of ketone bodies***

Plasma and heart β-OHB or AcAc levels were measured using the Ketone Body Assay Kit (Abnova, KA1630) according to the manufacturer’s instructions. The acetone concentrations were measured using the Goat Acetone Elisa Kit (MyBioSource, MBS9310393) according to the manufacturer’s instructions.

***Cell culture and treatments***

Rat cardiomyoblast cells (H9C2), mouse cardiac muscle cells (HL-1), human cardiomyocytes (HCM), and mouse embryonic fibroblasts (MEFs) cells were cultured in DMEM supplemented with 10% fetal bovine serum (FBS) (Biochrom), 100 units/ml penicillin (Invitrogen), and 100 mg/ml streptomycin (Invitrogen). In β-OHB treatment, 5 mM β-OHB were adding to the culture medium 24 h before harvesting. Deacetylase inhibitor treatments were carried out by adding Trichostatin A (TSA) (0.5 μM, final concentration) to the culture medium 24 h before harvesting. Hypoxia condition was induced by adding EC-Oxyrase (0.3 Unit/ml) to the culture medium for 6 h before harvesting.

***Cardiac fibroblast and cardiomyocyte isolation and culture***

Cardiac fibroblasts were collected from Sprague-Dawley neonate rat hearts by enzymatic digestion. Briefly, rats were administered 100 μl heparin (100 U/ml) via intraperitoneal injection and anesthetized with isoflurane. A part of the heart atrium was washed with PBS, and then equal volumes of 0.25% trypsin and 0.3% type I collagen were added before incubating the tissue slices in a 37°C water bath. Cells were collected every 10 min for a total of five collections, and the collected cells were filtered using a screen stencil and centrifuged at 1500 ×*g* for 5 min. Cardiomyocytes were pelleted by gravity (20 min), and the supernatant was collected and stored on ice. After enzymatic digestion, cardiac fibroblasts were cultured on plastic in DMEM (Gibco, Gaithersburg, MD, USA) supplemented with 100 U/ml penicillin, 100 mg/ml streptomycin, 2 mM l-glutamine, and 10% fetal calf serum (FCS).

***Plasmid construction and transfection***

*SIRT7* was cloned into the PRK7-Flag vector with a Flag tag at the N-terminus of the *SIRT7* gene. *HDAC1*, *HDAC2*, *HDAC3*, *HDAC4*, *HDAC5*, *HDAC6*, *HDAC7*, and *HDAC8* were each cloned into pcDNA3.1b-C-Flag with a Flag tag at the C-terminus of each gene. Primers and the restriction enzyme cutting sites used in plasmid construction are listed in Supplementary Table 1. Plasmid transfections were carried out by the polyethylenimine (PEI) or Lipofectamine 2000 (Invitrogen) methods. In the PEI transfection method, 500 μl of DMEM (serum-free medium) and the plasmid were placed in an empty Eppendorf tube and PEI (three times the concentration of plasmid) was added into the medium with vigorous shaking. The mixture was incubated for 15 min. Meanwhile, the cell culture medium was replaced with 2 ml of fresh 10% NCS medium. After 15 min, the mixture was added to the cells and fresh medium was replaced after 12 h. After 36 h, the transfection was completed and the cells were treated or harvested. In the Lipofectamine 2000 transfection method. DMEM (250 μl) was added to two clean Eppendorf tubes and 6 μl of Lipofectamine 2000 was added to one of the tubes and mixed for 5 min. The plasmid was added in the other tube and then added to the medium containing Lipofectamine 2000, mixed, and allowed to stand for 20 min. Meanwhile, the cell culture medium was replaced by serum-free medium as serum interferes with Lipofectamine 2000 transfection efficiency. At 6 h after the addition of the mixture to the cells, the medium was replaced with fresh normal medium and cells were collected 36 h later.

***Antibodies***

The antibodies against SIRT7 (#5360S, 1:1000), caspase 3 (#9662S, 1:2000), and LC3B (#3868S, 1:1000) were purchased from Cell Signaling Technology. The antibodies against GFM2 (#sc-514242, 1:200), POLRMT (#sc-365082, 1:200), TFAM (#sc-166965, 1:200), TFB2M (#sc-517095, 1:200), PINK1 (#sc-517353, 1:100), Parkin (#sc-133167, 1:200), and MRPL24 (#sc-393857, 1:500) were purchased from Santa Cruz Biotechnology. The antibodies against BDH1 (#ab193156, 1:1000), SCOT (#ab105320, 1:500), CACNA1H (#ab135974, 1:500), acetylated histone H3K9 (#ab4441, 1:3000), acetylated histone H3K14 (#ab52946, 1:1000), HDAC1 (#ab7028, 1:2000), HDAC2 (#ab12169, 1:2000), HDAC3 (#ab7030, 1:2000), HDAC4 (#ab12171, 1:1000), HDAC5 (#ab1439, 1:1000), HDAC6 (#ab1440, 1:1000), HDAC7 (#ab12174, 1:1000), and HDAC8 (#ab187139, 1:2000) were purchased from Abcam. The Histone 3.1 antibody (#P30266M, 1:10000) was from Abmart. The antibodies against type-I collagen (#C2456, 1:2000) and type-III collagen (#C7805, 1:4000) were obtained from Sigma-Aldrich. The α-SMA antibody (#BM0002, 1:500) was from Bosterbio. The CACNA2D2 antibody (#PA5-35132) was from Invitrogen. The antibody against pan-acetyllysine (1:500) was generated in our laboratory as described previously.^1^

***RNA interference***

Small RNA interference and stable knockdown were used in this study. In RNA interference, double-stranded siRNAs targeting *Sirt7* and *Hdac1-8*, respectively, were purchased from GenePharma and transfected into cells via RNAiMax (Invitrogen) according to manufacturer’s instructions.

Stable shRNA knockdown cells were generated by co-transfecting cells with pCMV-VSV-G, pCMV-Gag-Pol, and shRNA plasmids by the calcium phosphate method. DMEM containing 10% FBS was used to culture the cells for 6 h after the transfection. After 24 h of transfection, supernatant of the cultured medium was collected and used as retrovirus preparation to infect cells at a density of 10% confluence in 90 mm diameter dishes. Cells were re-infected 48 h after the initial infection using 5 μg/ml puromycin (Amresco) for selecting cells. All the sequences of siRNA/shRNA are listed in Supplementary Table 1. The knock-down efficiency was verified by quantitative PCR (q-PCR) or western blotting.

***Immunoprecipitation and immunoblotting***

Unless specified, collected cells were lysed in 0.5% NP-40 buffer containing 50 mM Tris-HCl (pH 7.5), 150 mM NaCl, 0.5% Nonidet P-40, and a mixture of protease inhibitors (Sigma-Aldrich). After centrifugation at 12,000 rpm and 4 °C for 15 min, the supernatant of the lysate was collected and incubated with anti-Flag M2 agarose or other antibody beads for 3 h at 4 °C. The beads were washed three times with lysis buffer before boiling with SDS loading buffer, followed by the standard immunoblotting procedures. Specifically, detection of acetylation by western blotting was achieved by using 50 mM Tris (pH 7.5) with 10% (v/v) Tween 20 and 1% peptone (OXOID) as blocking buffer and primary and secondary antibodies diluted in 50 mM Tris (pH 7.5) with 0.1% peptone.

***Chromatin immunoprecipitation (ChIP) assays***

ChIP assays were conducted using the EZ ChIP kit (Upstate). First, H9C2 cells treated with or without β-OHB, and heart samples from rats with various treatments (n = 3 rats per group) were crosslinked by 1% formaldehyde for 10 min. DNA was then sonicated into fragments with a mean length of 200 to 500 bp, and sheared chromatin was immunoprecipitated with antibodies against HDAC2, H3K9Ac, H3K14Ac, or non-specific rabbit IgG (Santa Cruz) overnight at 4 °C. The precipitated DNA fragments were then identified by PCR and quantified by real time q-PCR (primers are listed in Supplementary Table 1).

***Quantitative real time reverse transcription (qRT)-PCR***

RNA from cultured cells was prepared with TransZol (Trans Gen Biotech Co. Ltd.) and cDNA was synthesized from 5 µg RNA with TransScript First-Strand cDNA synthesis Super Mix (Trans Gen Biotech Co. Ltd.). Gene expression was determined by real time PCR using the iQ^TM^ SYBR Green SuperMix Kit (BIO-RAD) on an CFX96^TM^ Real-Time system (BIO-RAD). All data were normalized to *ACTB* (human) or *Actb* (rat) expression. All primer sequences are listed in Supplementary Table 1.

***In vitro HDAC activity assay***

Recombinant Flag-tagged HDAC proteins were expressed in HEK293T cells after transient transfection, purified with anti-Flag M2 agarose beads, and eluted with 3 × Flag peptide. Deacetylase assays were performed in 100 μL deacetylase buffer. The deacetylation reaction buffer included 4 mM MgCl_2_, 0.2 mM dithiothreitol, 50 mM Tris-HCl, and 0.5 mM phenylmethylsulfonyl fluoride (pH 8.5) plus 100 ng HDAC, a synthetic acetylated histone 3.1 peptide (sequence: ARTKQTARK[Ac]STGGKAPR) substrate, and different concentrations of β-OHB. Deacetylation reactions were conducted at 37°C and stopped by adding 25 μL stop solution (0.2 M HCl, 0.32 M acetic acid). The peptide was desalted by passing through a C18 ZipTip (Millipore), and the relative HDAC activity was measured using a matrix-assisted laser desorption ionization time-of-flight/time-of-flight mass spectrometer (SCIEX-5800).

***Histone extraction***

Cells were washed twice with PBS and resuspended in 200 μl of buffer A (10 mM HEPES (pH 7.9), 10 mM KCl, 1.5 mM MgCl_2_, 0.34 M sucrose, 10% glycerol, 1 mM DTT, 0.1% Triton X-100, and protease inhibitor mixture (Roche Molecular Biochemicals). After incubation for 5 min on ice, the nuclei were collected in the pellet by low speed centrifugation (1500 × *g*, 4 min, 4 °C). The nuclei were washed once with buffer A without 0.1% Triton X-100 and then lysed in 200 μl of buffer B (3 mM EDTA, 0.2 mM EGTA, 1 mM DTT, and protease inhibitor mixture). After 10 min incubation on ice, soluble histones were separated from chromatin by centrifugation (2000 × *g*, 4 min).

***Gas chromatography/mass spectrometry (GC/MS)***

For detection of metabolic intermediates, H9C2, HL-1 cells, or rat cardiac tissues were lysed by immediate addition of 1 mL 80% (v/v) prechilled (−80°C) methanol, followed by centrifugation at 12,000 × *g* at 4°C for 15 min. The supernatants were lyophilized followed by oximatation with 20 mg/ml methoxyamine hydrochloride in pyridine at 30°C for 60 min. The samples were then derivatized in 80 μL pyridine and 20 μL N-methyl-N-(tert-butyldimethylsilyl) trifluoroacetamide at 70°C for 30 min. Derivatized samples were filtered to remove insoluble particles, and 3 μL of each sample was subject to GC/MS analysis using an HP-5MS column (30 m × 0.25 mm × 0.25 μm) for separation on an Agilent 6890-5973 GC-MS system. The ions from pyruvate, citrate, α-ketoglutarate, succinate, fumarate, and lactate were monitored at *m/z* 174, 459, 346, 289, 287, and 261, respectively. For each measurement, triplicate measurements were obtained.

***Apoptosis assay***

FITC Annexin V Apoptosis Detection Kit I (BD Pharmingen) was used following the manufacturer’s guidelines. HL-1 cells were washed twice with cold PBS and then suspended in 1× Binding Buffer at a concentration of 1 × 10^6^ cells/ml. Then, 100 μl of the solution (1 × 10^5^ cells) was transferred to a 5 ml culture tube, to which 5 μl of FITC Annexin V and 5 μl propidium iodide were added. The cells were gently vortexed and incubated for 15 min at room temperature (25 °C) in the dark. After adding 400 μl of 1X Binding Buffer to each tube, apoptosis was analyzed using an Accuri C6 flow cytometer (BD Biosciences) within 1 h.

***TUNEL assay***

Rat atrial tissues were paraffin-embedded, sliced, soaked in xylene for 30 min, deparaffinized, and rehydrated gradiently. After washing with PBS three times, the sections were fixed with 4% paraformaldehyde and permeabilized with 0.1% permeabilization solution. Then, 100 μl of TUNEL staining solution was added and incubated at room temperature (25 °C) for 1 h. The sections were washed with PBS for 5 min, and staining was observed under a fluorescence microscope. The apoptotic rate was calculated using the following equation: Number of green-stained cells / Number of blue-stained cells × 100%.

***Measurements of mitochondrial mass and mtDNA/nDNA***

For mitochondrial mass measurements, after appropriate treatments, cells were harvested by trypsinization and washed twice in PBS. Cells were then resuspended in 100 μl of PBS containing 100 nM MitoTracker Green (MTG; Invitrogen) and incubated at 37 °C for 30 min. Cells were then washed twice in PBS and analyzed by flow cytometry in a flow cytometer (BD Calibur) using a 488 nm argon excitation laser. Results presented for each cell group represent the means ± S.E. of the fluorescence intensity of three independent experiments. Moreover, we used the ratio of the mitochondrial DNA to nuclear DNA (mtDNA/nDNA) to indicate the mitochondrial levels. The primers used for quantifying mitochondrial DNA and nuclear DNA are shown in Supplementary Table 1.

***Mitochondrial DNA damage quantification***

For mitochondrial DNA damage quantification, a LongAmp PCR-Based mitochondrial DNA damage assay was performed.^2^ Briefly, total rat cardiomyocytes DNA was extracted by using a QIAamp DNA Mini Kit (QIAGEN, Cat No. 51304), large mitochondrial PCR product and small mitochondrial PCR product was amplificated by using the LongAmp® Hot Start Taq DNA Polymerase (NEB, Cat No. M0534) and PCR product fluorescence was quantified using the Quant-iT™ PicoGreen™ dsDNA Assay Kit (Thermo, Cat No. P7589), the large mitochondrial PCR product fluorescence values was normalized using the small mitochondrial PCR product for copy number differences, and the lesion frequency per 10 kb was calculated using a method previously published.^3^ The primers used for in this assay are shown in Supplementary Table 1.

***Measurements of oxygen consumption***

HCM or H9C2 cells or rat cardiomyocytes were treated with or without 5 mM β-OHB. After 72 h of culture, cells or cardiomyocytes were washed with PBS and centrifuged before seeding into XFe24 Cell Culture Microplates (Seahorse Bioscience, Billerica, MA, USA) at a density of 1.5–4 × 10^4^ cells/well. The Seahorse Bioscience assay medium contains 4500 mg/L glucose, 4 mM l-glutamine, and 1 mM sodium pyruvate. The oxygen consumption rate (OCR) at baseline and following treatment with the mitochondrial inhibitor oligomycin (1 μM), mitochondrial uncoupler FCCP (1 μM), or respiratory chain inhibitors rotenone and antimycin A (1 μM) were determined using a Seahorse Bioscience XF24 instrument following the manufacturer’s instructions.

***Immunofluorescence***

To detect the expression of SIRT7 and manganese SOD2 (MnSOD2) protein, immunofluorescence was carried out in atrial tissue samples. Briefly, paraffin-embedded heart sections were first incubated for 30 min at room temperature with 2% BSA to reduce nonspecific binding, followed by overnight incubation at 4°C with antibodies for rabbit polyclonal anti-SIRT7 (1:50) and mouse anti-MnSOD2 (1:100). After washing in phosphate-buffered saline (PBS), sections were incubated in the dark for 3 h at 37°C with fluorescein isothiocyanate-conjugated goat anti-rabbit IgG (1:50; Boster) and tetramethylrhodamine isothiocyanate-conjugated goat anti-mouse IgG (1:50; Boster). After washing with PBS, the slides were mounted with 50% glycerol and 50% PBS. Cover slips were mounted on to microscope slides using fluorescence mounting medium (Dako) and observed under an inverted fluorescence microscope (Olympus).

**Reference**

1. Guan KL, Yu W, Lin Y, Xiong Y, Zhao S. Generation of acetyllysine antibodies and affinity enrichment of acetylated peptides. *NAT PROTOC.* 5(9):1583-95. doi: 10.1038/nprot.2010.117 (2010).
2. Laurie H. Sanders, Jeremy P. Rouanet, Evan H. Howlett, Tess C. Leuthner, John P. Rooney, J. Timothy Greenamyre, Joel N. Meyer, Newly Revised Quantitative PCR-Based Assay for Mitochondrial and Nuclear DNA Damage. *CURR PROTOC TOXICOL* 76(1):e50 (2018).
3. Gonzalez-Hunt CP, Rooney JP, Ryde IT, Anbalagan C, Joglekar R, Meyer JN, PCR-Based Analysis of Mitochondrial DNA Copy Number, Mitochondrial DNA Damage, and Nuclear DNA Damage. *CURR PROTOC TOXICOL*. 67:20.11.1-20.11.25 (2016).
