# Supplementary material for: Ketogenic diets inhibit mitochondrial biogenesis and induce cardiac fibrosis
Source: Signal Transduct Target Ther. 2021 Feb 9;6:54. doi: 10.1038/s41392-020-00411-4 (PMC7870678; doi:10.1038/s41392-020-00411-4)
Supplement: Supplementary file 3 — Supplementary Figures and Table [file 41392_2020_411_MOESM3_ESM.pptx]

## Slide 1
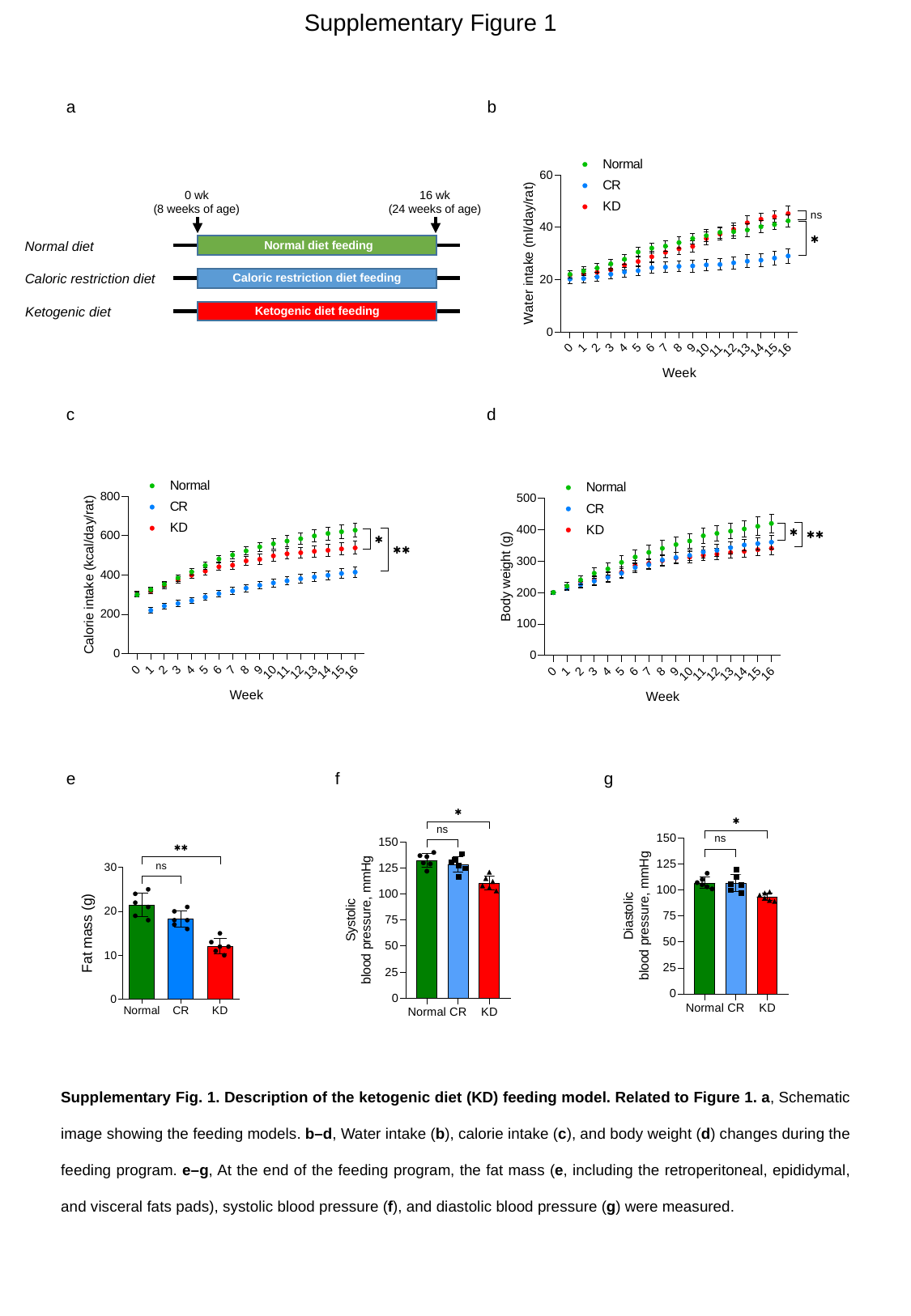

Supplementary Figure 1
a
b
0 wk
(8 weeks of age)
16 wk
(24 weeks of age)
Normal diet feeding
Normal diet
Caloric restriction diet feeding
Caloric restriction diet
Ketogenic diet
Ketogenic diet feeding
c
d
e
f
g
Supplementary Fig. 1. Description of the ketogenic diet (KD) feeding model. Related to Figure 1. a, Schematic image showing the feeding models. b–d, Water intake (b), calorie intake (c), and body weight (d) changes during the feeding program. e–g, At the end of the feeding program, the fat mass (e, including the retroperitoneal, epididymal, and visceral fats pads), systolic blood pressure (f), and diastolic blood pressure (g) were measured.

## Slide 2
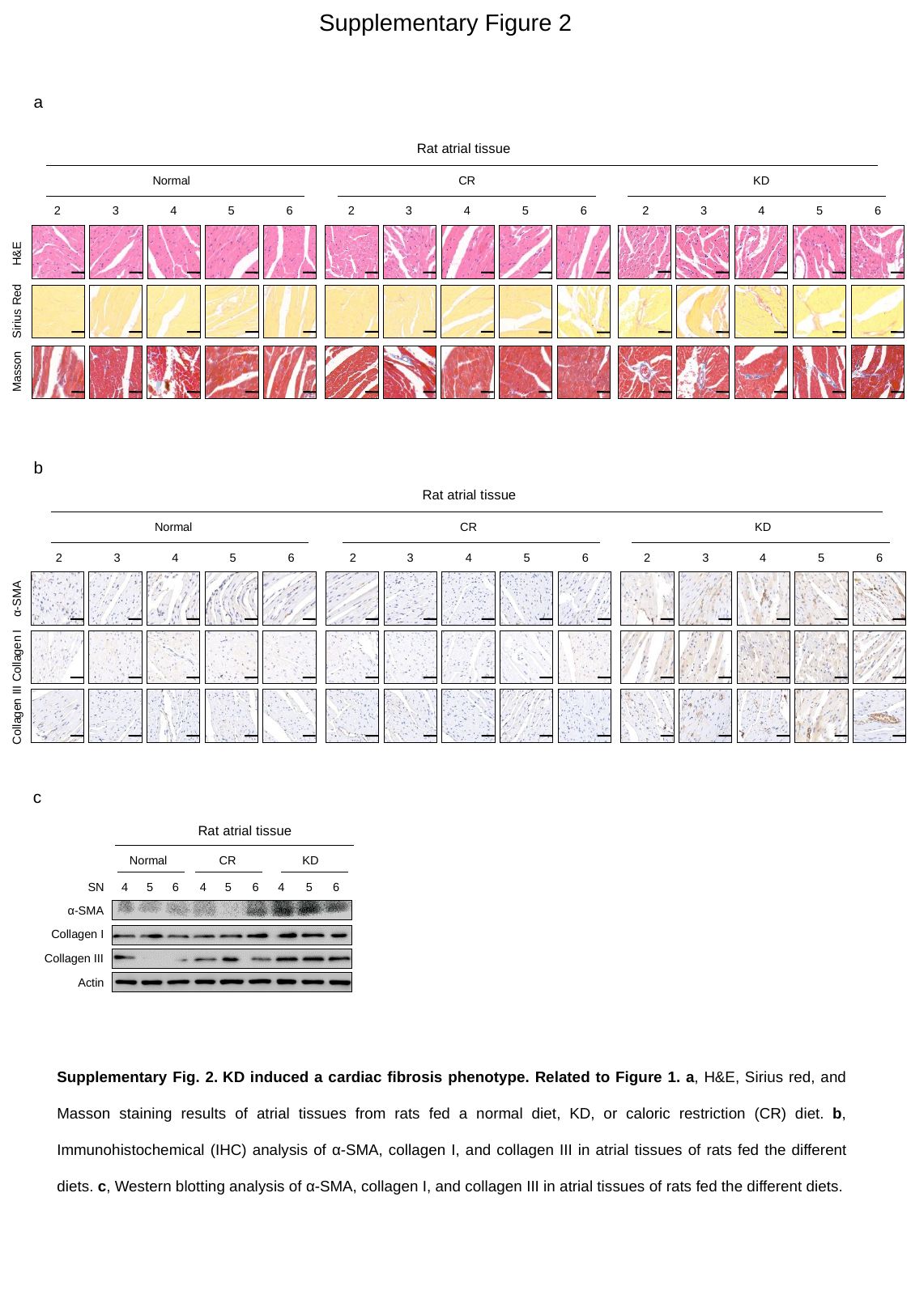

Supplementary Figure 2
a
Rat atrial tissue
CR
KD
Normal
2
3
4
5
6
2
3
4
5
6
2
3
4
5
6
H&E
Sirius Red
Masson
b
Rat atrial tissue
CR
KD
Normal
2
3
4
5
6
2
3
4
5
6
2
3
4
5
6
α-SMA
Collagen I
Collagen III
c
Rat atrial tissue
Normal
CR
KD
4
5
6
4
5
6
4
5
6
SN
α-SMA
Collagen I
Collagen III
Actin
Supplementary Fig. 2. KD induced a cardiac fibrosis phenotype. Related to Figure 1. a, H&E, Sirius red, and Masson staining results of atrial tissues from rats fed a normal diet, KD, or caloric restriction (CR) diet. b, Immunohistochemical (IHC) analysis of α-SMA, collagen I, and collagen III in atrial tissues of rats fed the different diets. c, Western blotting analysis of α-SMA, collagen I, and collagen III in atrial tissues of rats fed the different diets.

## Slide 3
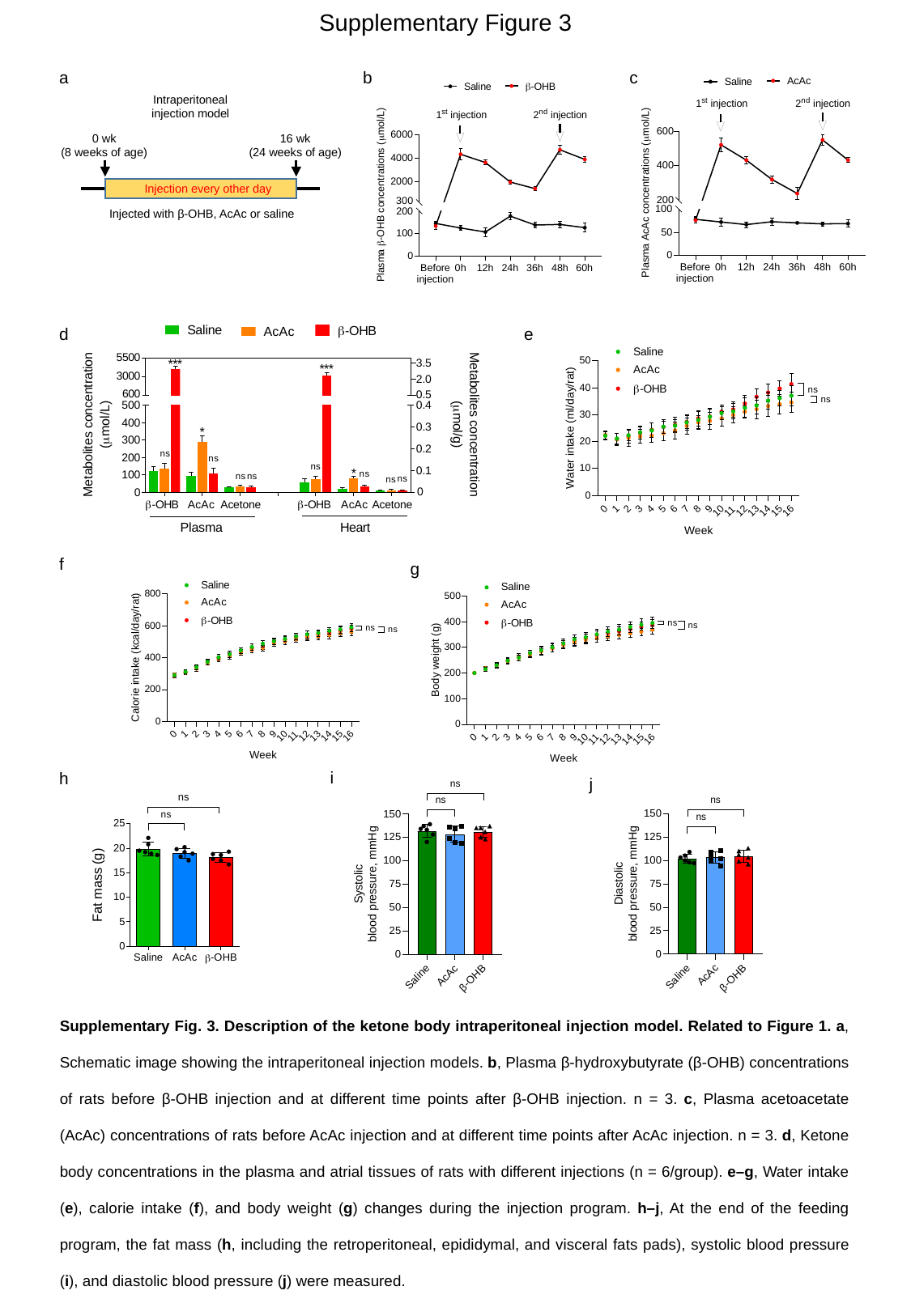

Supplementary Figure 3
a
b
c
Intraperitoneal injection model
0 wk
(8 weeks of age)
16 wk
(24 weeks of age)
Injection every other day
Injected with β-OHB, AcAc or saline
d
e
f
g
i
h
j
Supplementary Fig. 3. Description of the ketone body intraperitoneal injection model. Related to Figure 1. a, Schematic image showing the intraperitoneal injection models. b, Plasma β-hydroxybutyrate (β-OHB) concentrations of rats before β-OHB injection and at different time points after β-OHB injection. n = 3. c, Plasma acetoacetate (AcAc) concentrations of rats before AcAc injection and at different time points after AcAc injection. n = 3. d, Ketone body concentrations in the plasma and atrial tissues of rats with different injections (n = 6/group). e–g, Water intake (e), calorie intake (f), and body weight (g) changes during the injection program. h–j, At the end of the feeding program, the fat mass (h, including the retroperitoneal, epididymal, and visceral fats pads), systolic blood pressure (i), and diastolic blood pressure (j) were measured.

## Slide 4
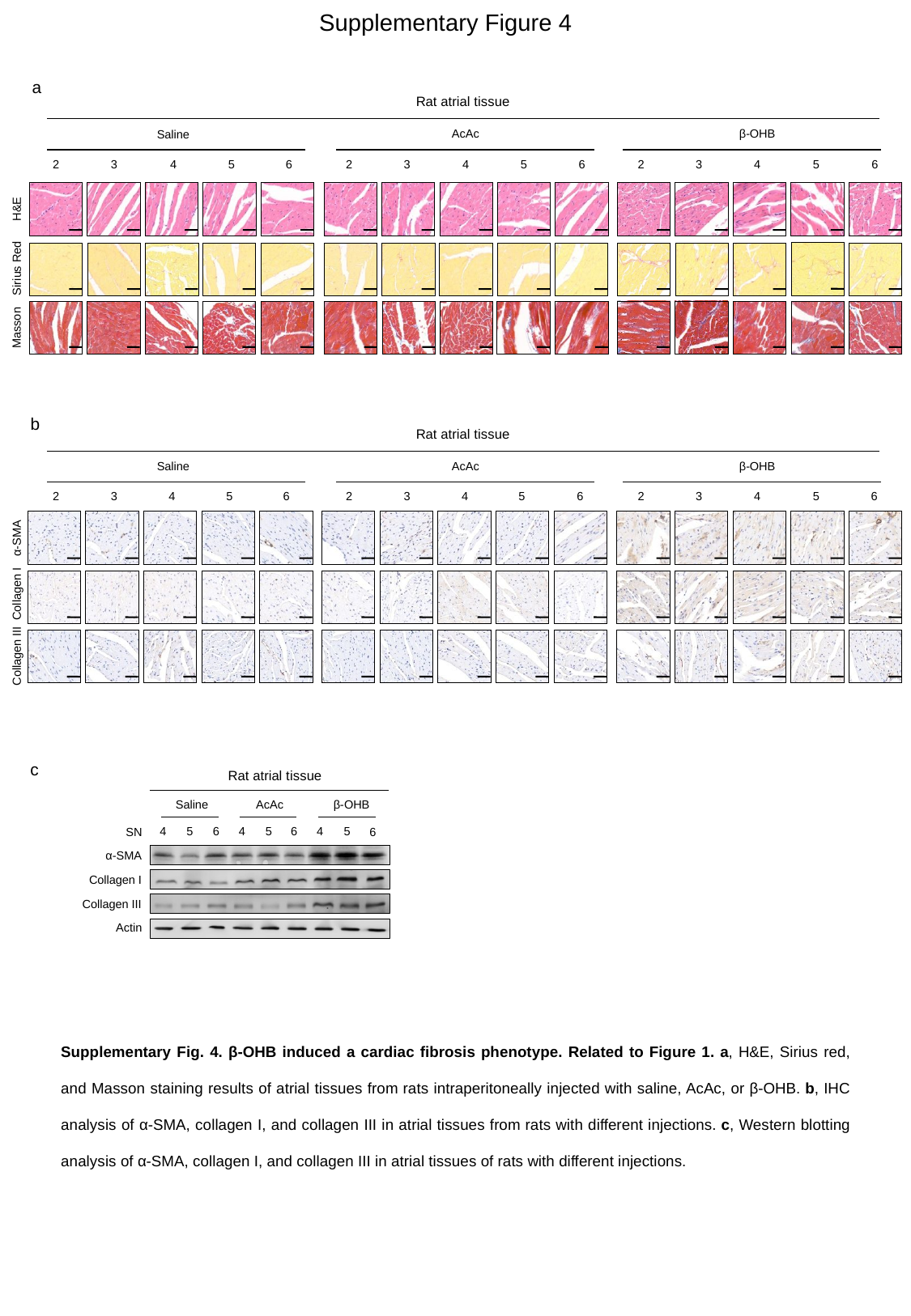

Supplementary Figure 4
a
Rat atrial tissue
AcAc
β-OHB
Saline
2
3
4
5
6
2
3
4
5
6
2
3
4
5
6
H&E
Sirius Red
Masson
b
Rat atrial tissue
AcAc
β-OHB
Saline
2
3
4
5
6
2
3
4
5
6
2
3
4
5
6
α-SMA
Collagen I
Collagen III
c
Rat atrial tissue
Saline
AcAc
β-OHB
4
5
6
4
5
6
4
5
6
SN
α-SMA
Collagen I
Collagen III
Actin
Supplementary Fig. 4. β-OHB induced a cardiac fibrosis phenotype. Related to Figure 1. a, H&E, Sirius red, and Masson staining results of atrial tissues from rats intraperitoneally injected with saline, AcAc, or β-OHB. b, IHC analysis of α-SMA, collagen I, and collagen III in atrial tissues from rats with different injections. c, Western blotting analysis of α-SMA, collagen I, and collagen III in atrial tissues of rats with different injections.

## Slide 5
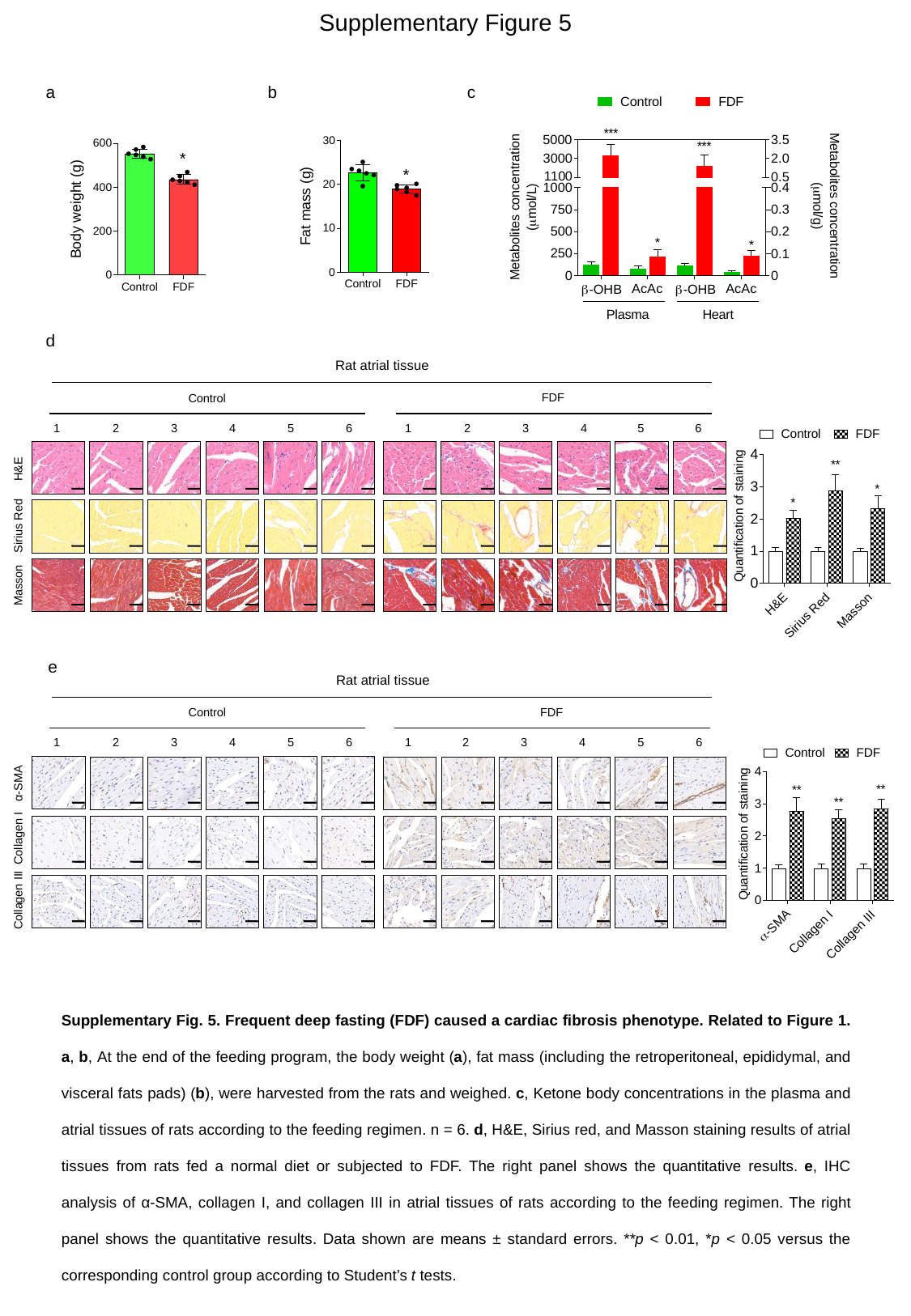

Supplementary Figure 5
a
b
c
d
Rat atrial tissue
FDF
Control
1
2
3
4
5
6
1
2
3
4
5
6
H&E
Sirius Red
Masson
e
Rat atrial tissue
FDF
Control
1
2
3
4
5
6
1
2
3
4
5
6
α-SMA
Collagen I
Collagen III
Supplementary Fig. 5. Frequent deep fasting (FDF) caused a cardiac fibrosis phenotype. Related to Figure 1. a, b, At the end of the feeding program, the body weight (a), fat mass (including the retroperitoneal, epididymal, and visceral fats pads) (b), were harvested from the rats and weighed. c, Ketone body concentrations in the plasma and atrial tissues of rats according to the feeding regimen. n = 6. d, H&E, Sirius red, and Masson staining results of atrial tissues from rats fed a normal diet or subjected to FDF. The right panel shows the quantitative results. e, IHC analysis of α-SMA, collagen I, and collagen III in atrial tissues of rats according to the feeding regimen. The right panel shows the quantitative results. Data shown are means ± standard errors. **p < 0.01, *p < 0.05 versus the corresponding control group according to Student’s t tests.

## Slide 6
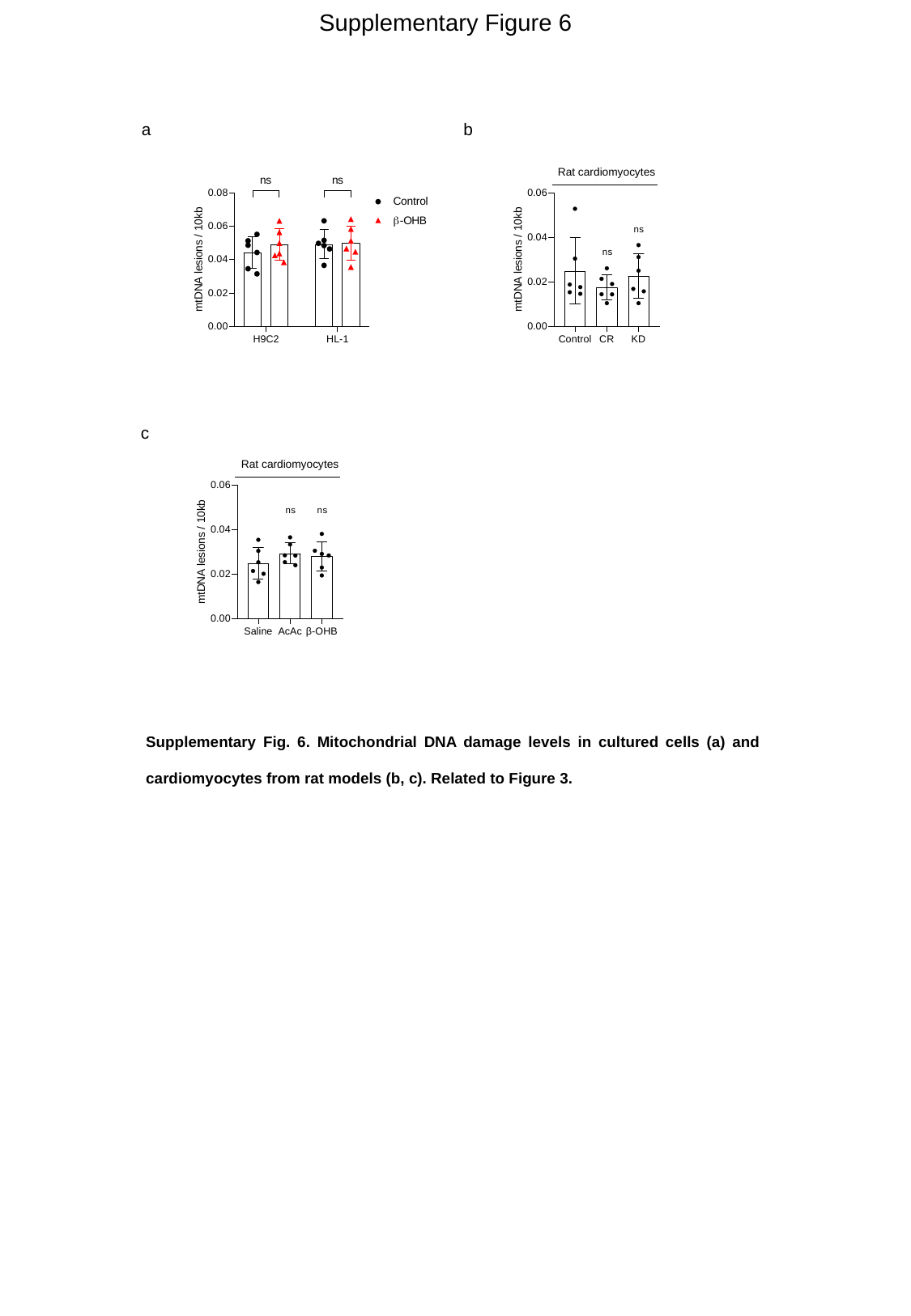

Supplementary Figure 6
a
b
c
Supplementary Fig. 6. Mitochondrial DNA damage levels in cultured cells (a) and cardiomyocytes from rat models (b, c). Related to Figure 3.

## Slide 7
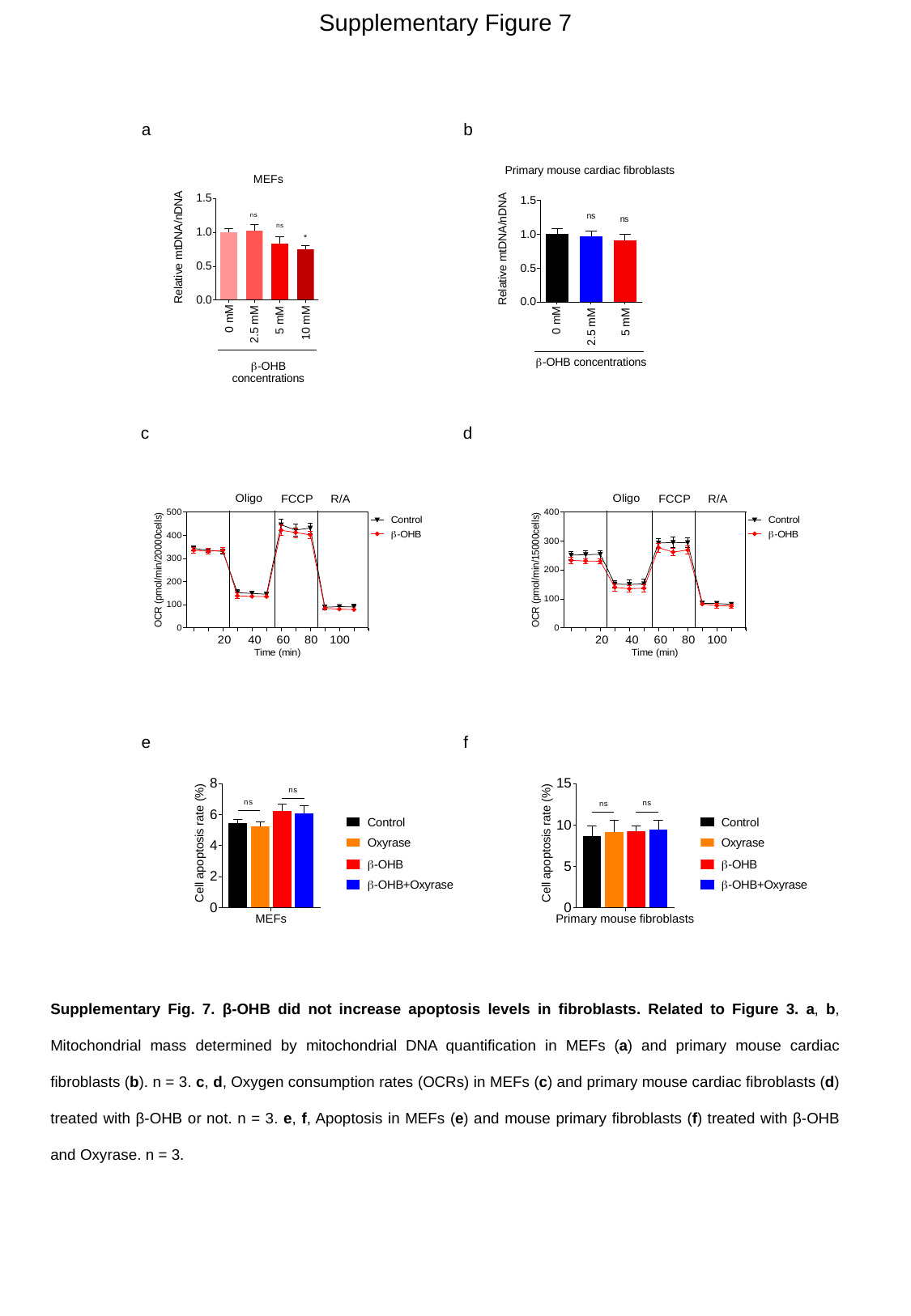

Supplementary Figure 7
a
b
c
d
e
f
Supplementary Fig. 7. β-OHB did not increase apoptosis levels in fibroblasts. Related to Figure 3. a, b, Mitochondrial mass determined by mitochondrial DNA quantification in MEFs (a) and primary mouse cardiac fibroblasts (b). n = 3. c, d, Oxygen consumption rates (OCRs) in MEFs (c) and primary mouse cardiac fibroblasts (d) treated with β-OHB or not. n = 3. e, f, Apoptosis in MEFs (e) and mouse primary fibroblasts (f) treated with β-OHB and Oxyrase. n = 3.

## Slide 8
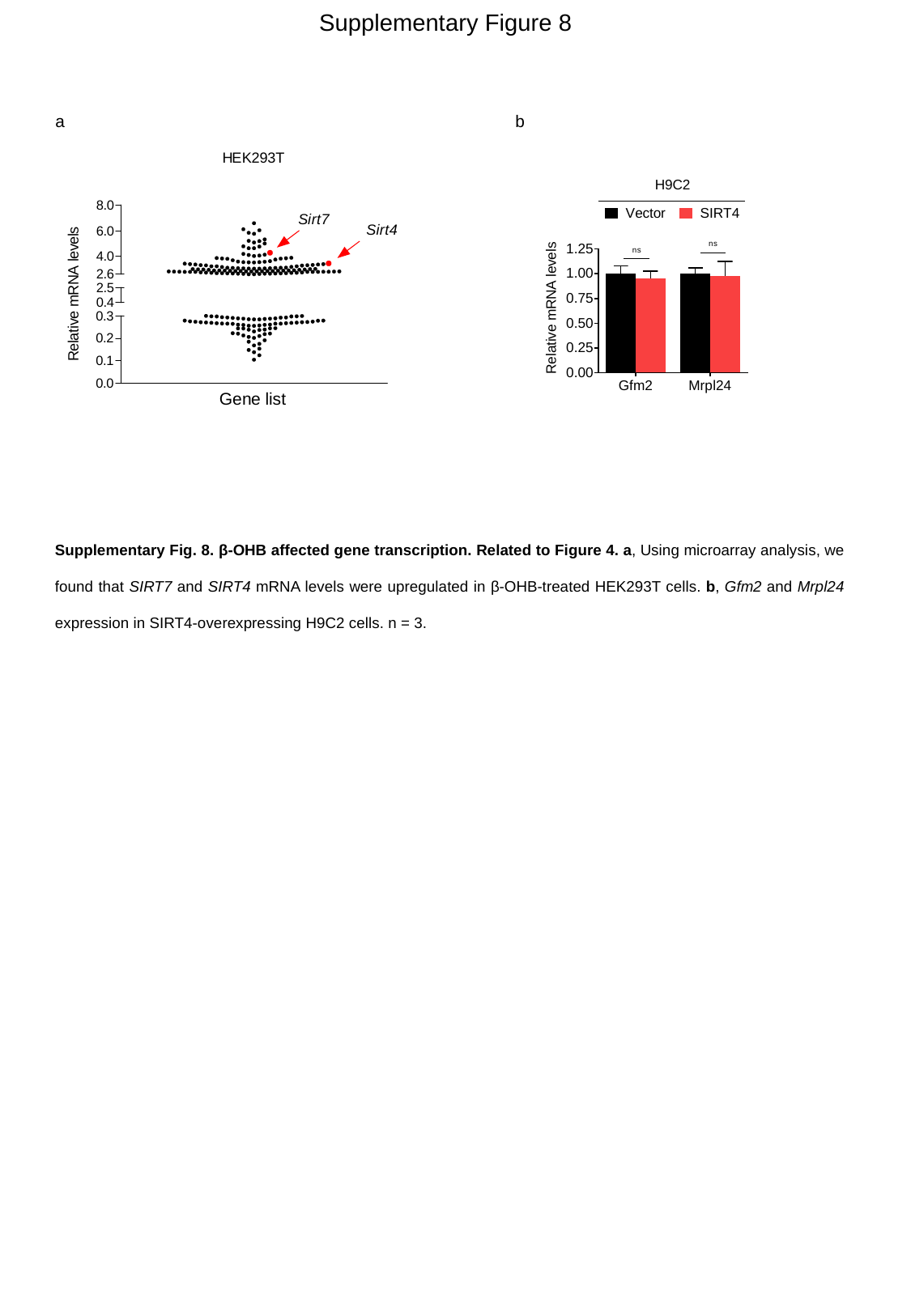

Supplementary Figure 8
b
a
Supplementary Fig. 8. β-OHB affected gene transcription. Related to Figure 4. a, Using microarray analysis, we found that SIRT7 and SIRT4 mRNA levels were upregulated in β-OHB-treated HEK293T cells. b, Gfm2 and Mrpl24 expression in SIRT4-overexpressing H9C2 cells. n = 3.

## Slide 9
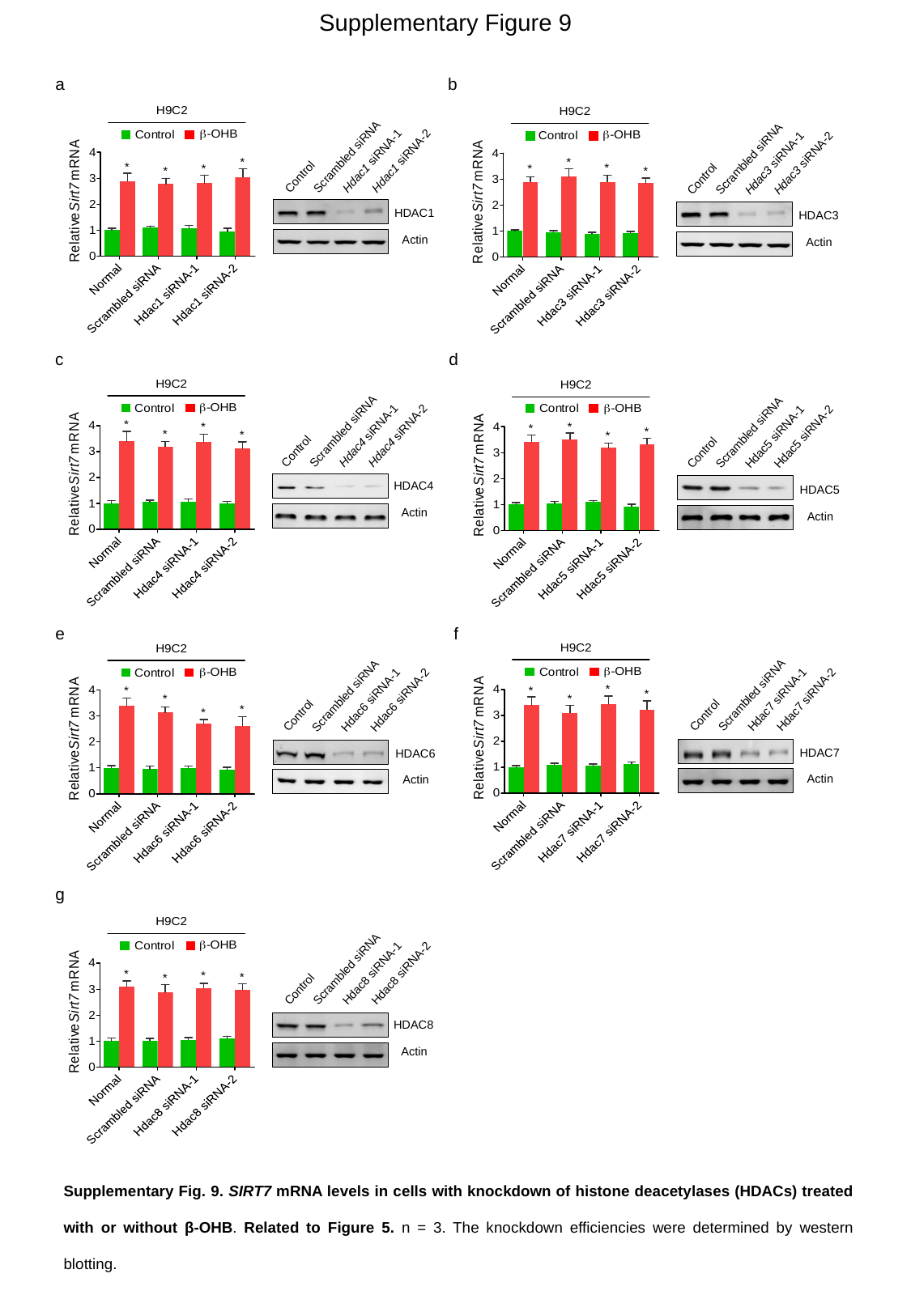

Supplementary Figure 9
a
b
Scrambled siRNA
Hdac1 siRNA-1
Hdac1 siRNA-2
Control
HDAC1
Actin
Scrambled siRNA
Hdac3 siRNA-1
Hdac3 siRNA-2
Control
HDAC3
Actin
c
d
Scrambled siRNA
Hdac4 siRNA-1
Hdac4 siRNA-2
Control
HDAC4
Actin
Scrambled siRNA
Hdac5 siRNA-1
Hdac5 siRNA-2
Control
HDAC5
Actin
e
f
Scrambled siRNA
Scrambled siRNA
Hdac7 siRNA-1
Hdac7 siRNA-2
Hdac6 siRNA-1
Hdac6 siRNA-2
Control
Control
HDAC7
HDAC6
Actin
Actin
g
Scrambled siRNA
Hdac8 siRNA-1
Hdac8 siRNA-2
Control
HDAC8
Actin
Supplementary Fig. 9. SIRT7 mRNA levels in cells with knockdown of histone deacetylases (HDACs) treated with or without β-OHB. Related to Figure 5. n = 3. The knockdown efficiencies were determined by western blotting.

## Slide 10
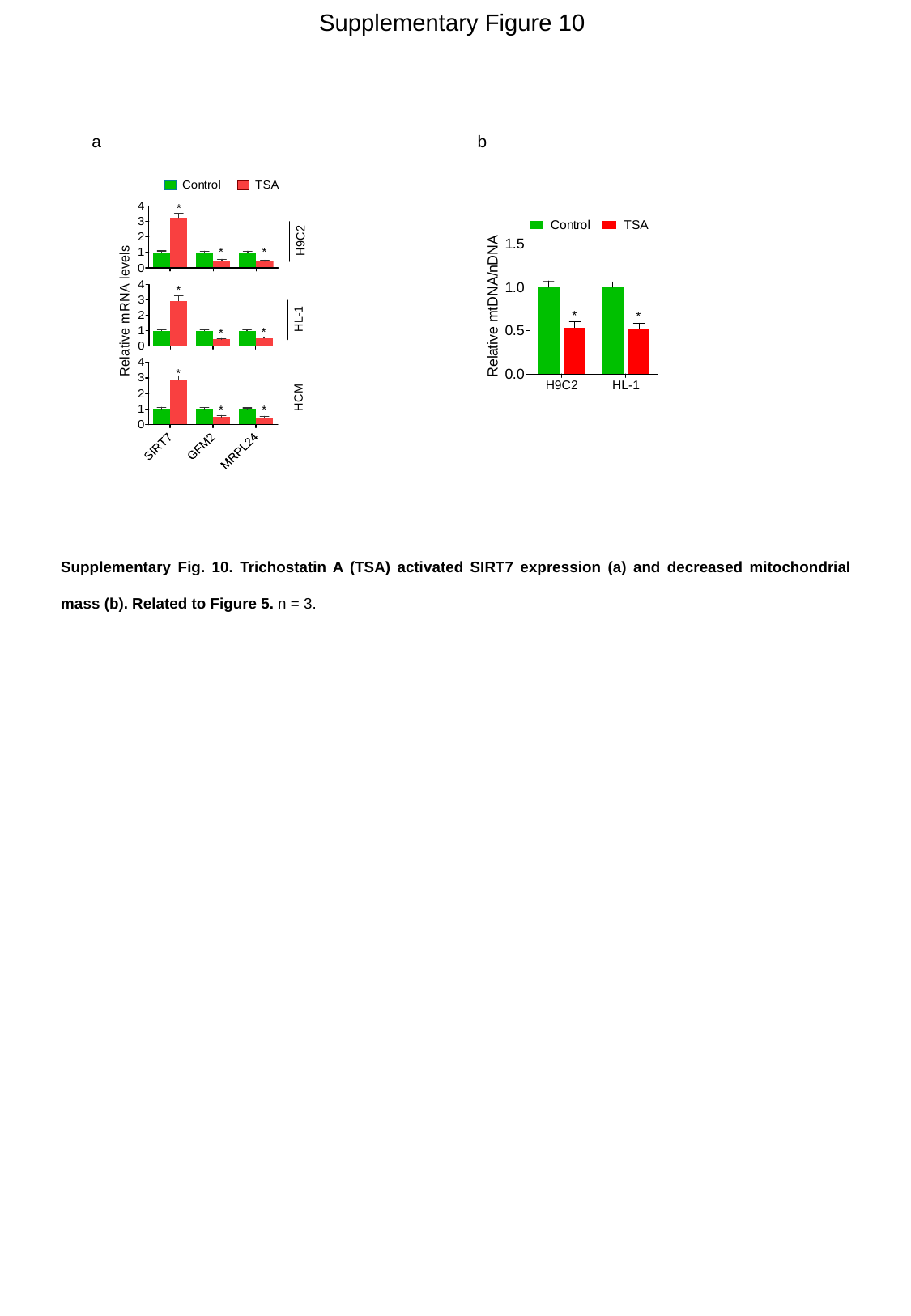

Supplementary Figure 10
a
b
Supplementary Fig. 10. Trichostatin A (TSA) activated SIRT7 expression (a) and decreased mitochondrial mass (b). Related to Figure 5. n = 3.

## Slide 11
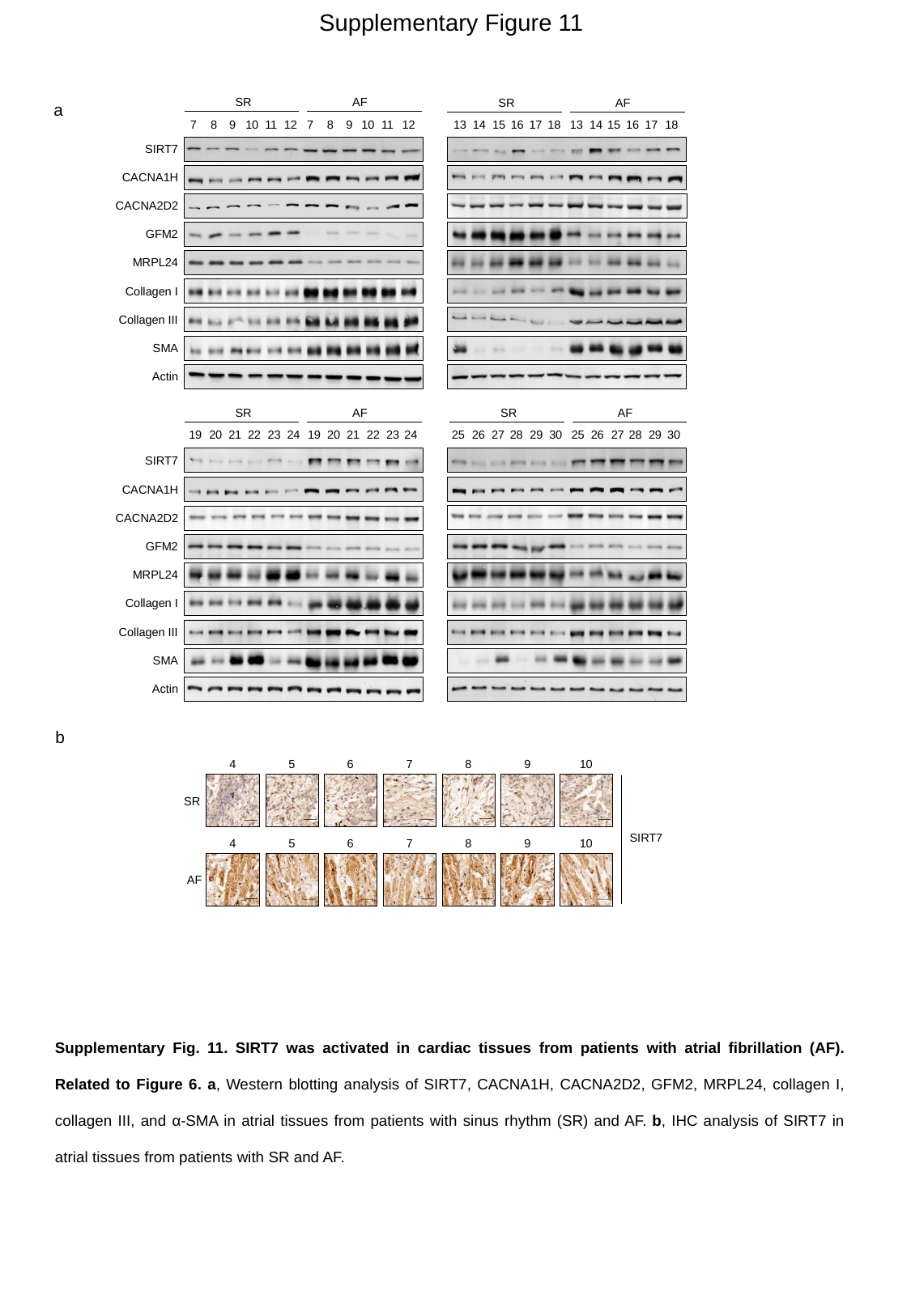

Supplementary Figure 11
SR
AF
SR
AF
7
8
9
10
11
12
7
8
9
10
11
12
13
14
15
16
17
18
13
14
15
16
17
18
SIRT7
CACNA1H
CACNA2D2
GFM2
MRPL24
Collagen I
Collagen III
SMA
Actin
SR
AF
SR
AF
19
20
21
22
23
24
19
20
21
22
23
24
25
26
27
28
29
30
25
26
27
28
29
30
SIRT7
CACNA1H
CACNA2D2
GFM2
MRPL24
Collagen I
Collagen III
SMA
Actin
a
b
4
5
6
7
8
9
10
SR
4
5
6
7
8
9
10
SIRT7
AF
Supplementary Fig. 11. SIRT7 was activated in cardiac tissues from patients with atrial fibrillation (AF). Related to Figure 6. a, Western blotting analysis of SIRT7, CACNA1H, CACNA2D2, GFM2, MRPL24, collagen I, collagen III, and α-SMA in atrial tissues from patients with sinus rhythm (SR) and AF. b, IHC analysis of SIRT7 in atrial tissues from patients with SR and AF.

## Slide 12
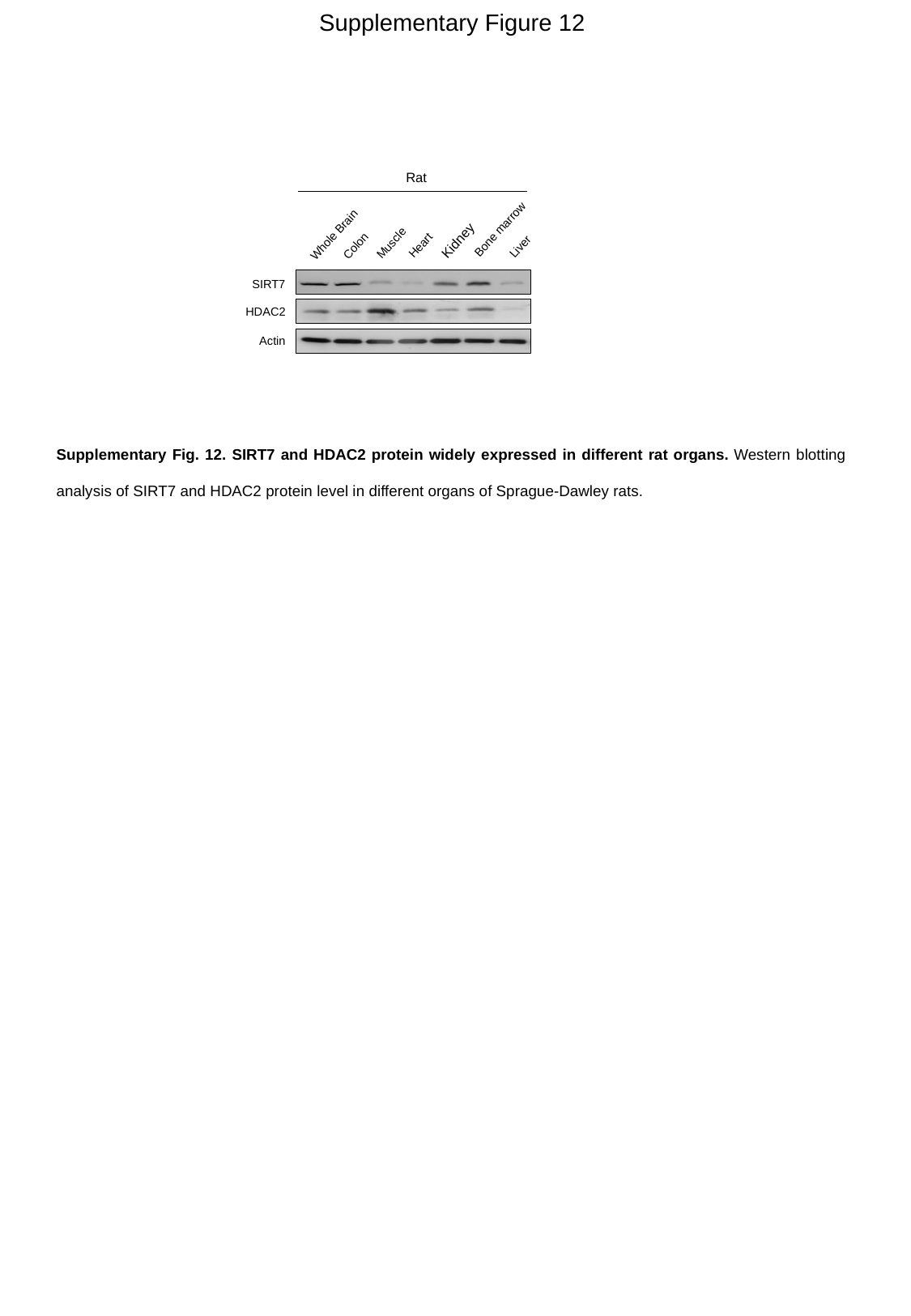

Supplementary Figure 12
Rat
Muscle
Kidney
Bone marrow
Heart
Liver
Whole Brain
Colon
SIRT7
HDAC2
Actin
Supplementary Fig. 12. SIRT7 and HDAC2 protein widely expressed in different rat organs. Western blotting analysis of SIRT7 and HDAC2 protein level in different organs of Sprague-Dawley rats.

## Slide 13
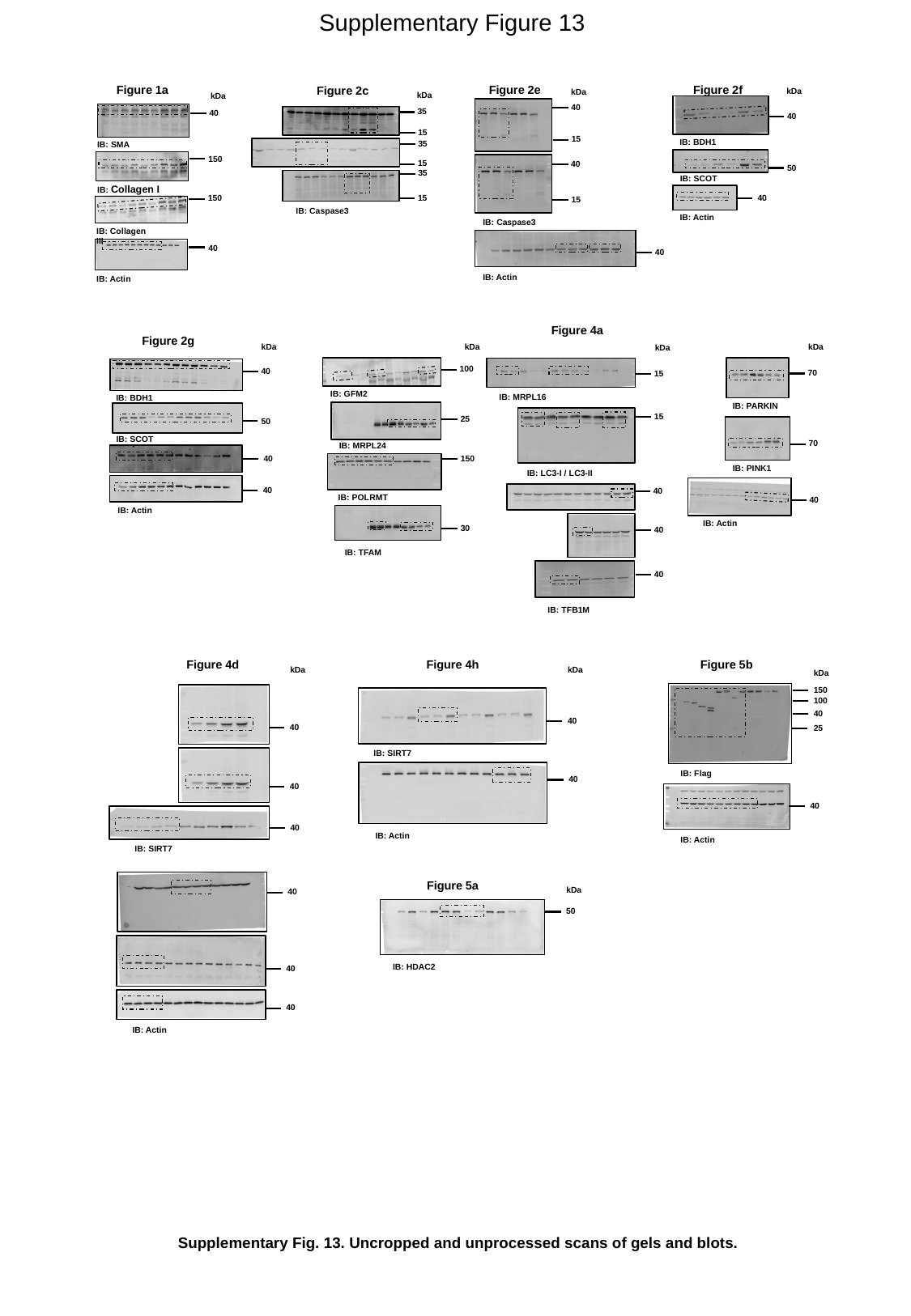

Supplementary Figure 13
Figure 2f
Figure 1a
Figure 2e
Figure 2c
kDa
kDa
kDa
kDa
40
35
40
40
15
15
IB: BDH1
35
IB: SMA
150
15
40
50
35
IB: SCOT
IB: Collagen I
15
40
150
15
IB: Caspase3
IB: Actin
IB: Caspase3
IB: Collagen III
40
40
IB: Actin
IB: Actin
Figure 4a
Figure 2g
kDa
kDa
kDa
kDa
100
40
70
15
IB: GFM2
IB: MRPL16
IB: BDH1
IB: PARKIN
15
25
50
IB: SCOT
70
IB: MRPL24
40
150
IB: PINK1
IB: LC3-I / LC3-II
40
40
IB: POLRMT
40
IB: Actin
IB: Actin
30
40
IB: TFAM
40
IB: TFB1M
Figure 4d
Figure 4h
Figure 5b
kDa
kDa
kDa
150
100
40
40
40
25
IB: SIRT7
IB: Flag
40
40
40
40
IB: Actin
IB: Actin
IB: SIRT7
Figure 5a
kDa
40
50
IB: HDAC2
40
40
IB: Actin
Supplementary Fig. 13. Uncropped and unprocessed scans of gels and blots.

## Slide 14
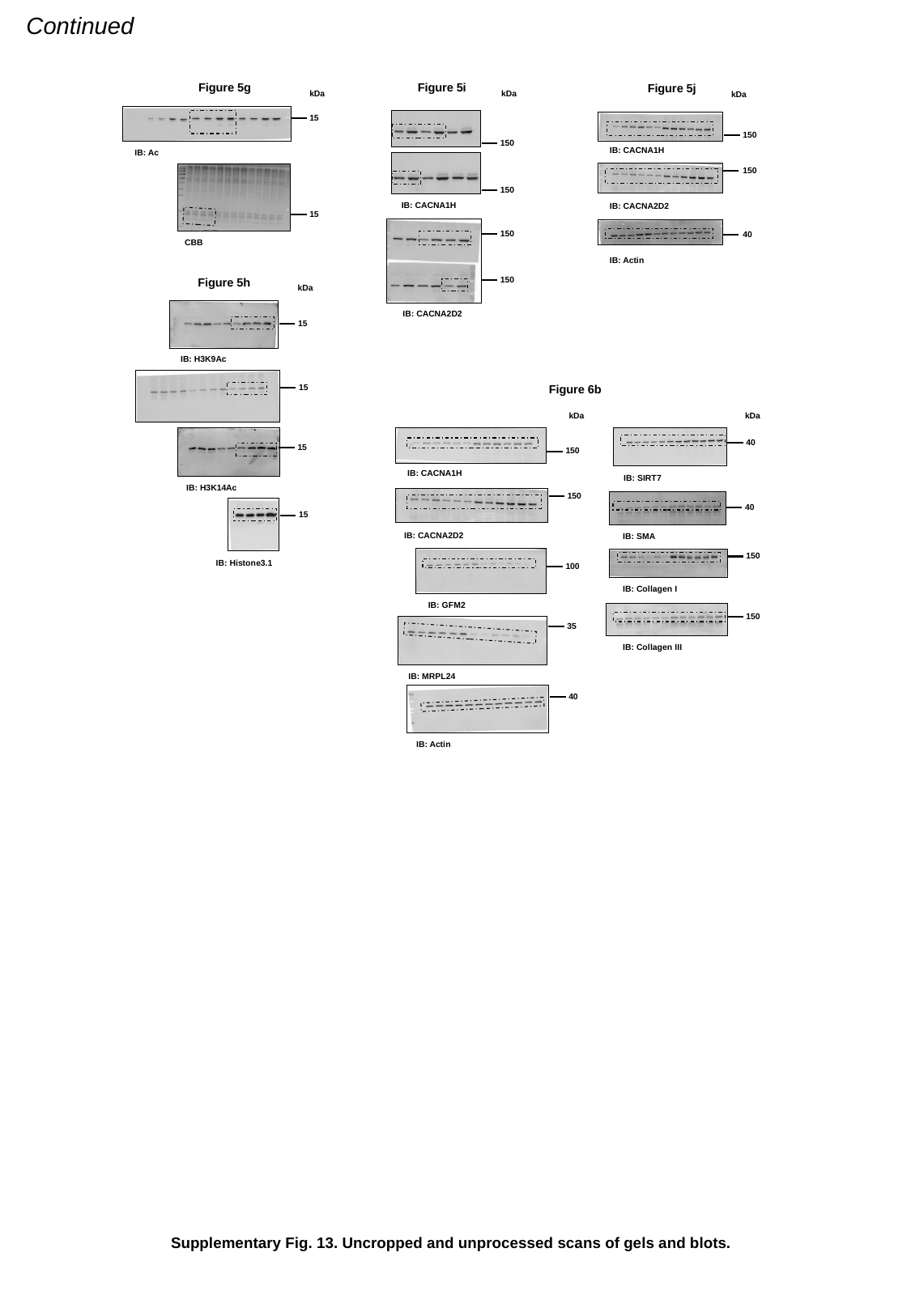

Continued
Figure 5i
Figure 5g
Figure 5j
kDa
kDa
kDa
15
150
150
IB: CACNA1H
IB: Ac
150
150
IB: CACNA1H
IB: CACNA2D2
15
150
40
CBB
IB: Actin
Figure 5h
150
kDa
IB: CACNA2D2
15
IB: H3K9Ac
Figure 6b
15
kDa
kDa
40
15
150
IB: CACNA1H
IB: SIRT7
IB: H3K14Ac
150
40
15
IB: CACNA2D2
IB: SMA
150
IB: Histone3.1
100
IB: Collagen I
IB: GFM2
150
35
IB: Collagen III
IB: MRPL24
40
IB: Actin
Supplementary Fig. 13. Uncropped and unprocessed scans of gels and blots.

## Slide 15
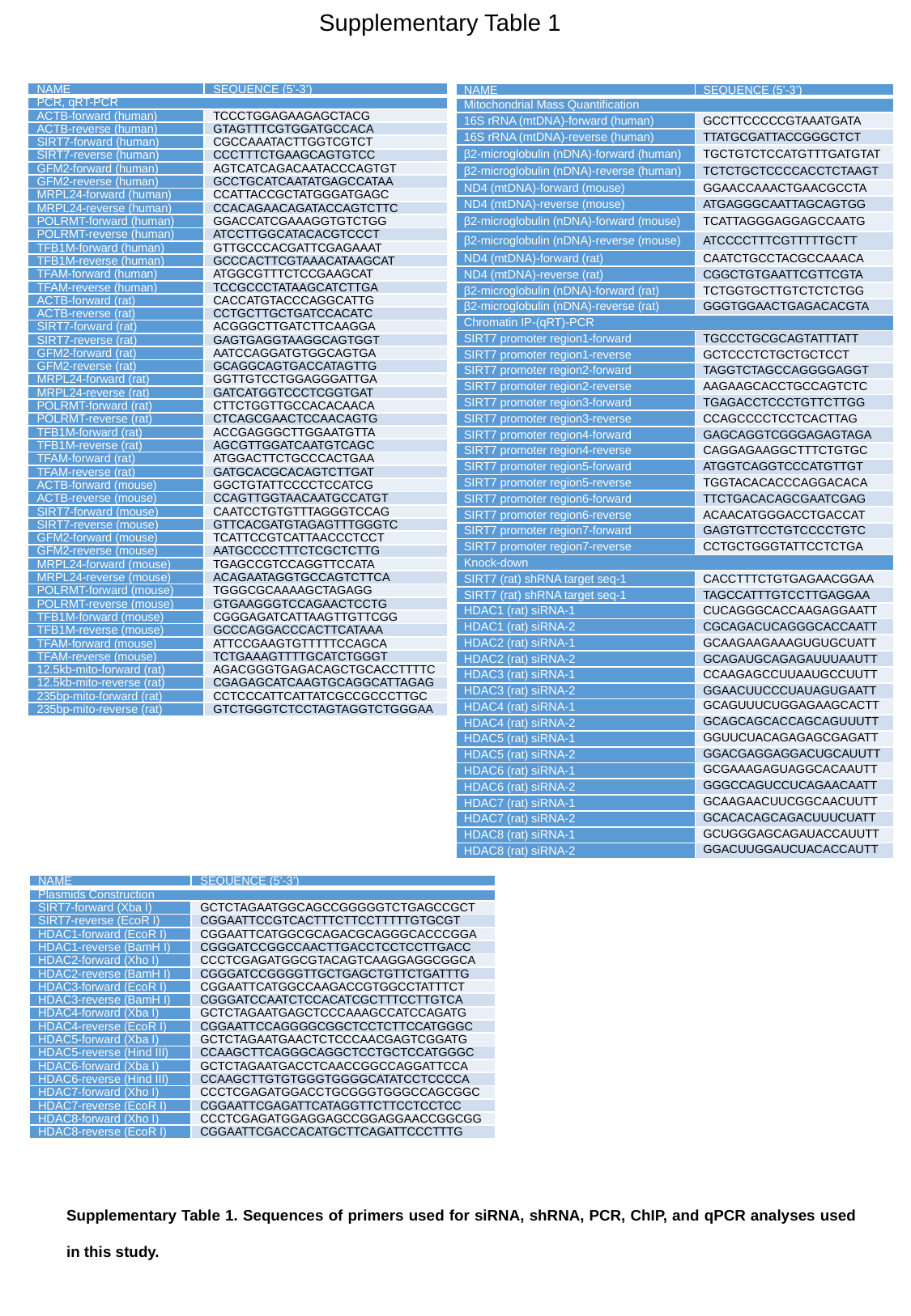

Supplementary Table 1
| NAME | SEQUENCE (5’-3’) |
| --- | --- |
| PCR, qRT-PCR | |
| ACTB-forward (human) | TCCCTGGAGAAGAGCTACG |
| ACTB-reverse (human) | GTAGTTTCGTGGATGCCACA |
| SIRT7-forward (human) | CGCCAAATACTTGGTCGTCT |
| SIRT7-reverse (human) | CCCTTTCTGAAGCAGTGTCC |
| GFM2-forward (human) | AGTCATCAGACAATACCCAGTGT |
| GFM2-reverse (human) | GCCTGCATCAATATGAGCCATAA |
| MRPL24-forward (human) | CCATTACCGCTATGGGATGAGC |
| MRPL24-reverse (human) | CCACAGAACAGATACCAGTCTTC |
| POLRMT-forward (human) | GGACCATCGAAAGGTGTCTGG |
| POLRMT-reverse (human) | ATCCTTGGCATACACGTCCCT |
| TFB1M-forward (human) | GTTGCCCACGATTCGAGAAAT |
| TFB1M-reverse (human) | GCCCACTTCGTAAACATAAGCAT |
| TFAM-forward (human) | ATGGCGTTTCTCCGAAGCAT |
| TFAM-reverse (human) | TCCGCCCTATAAGCATCTTGA |
| ACTB-forward (rat) | CACCATGTACCCAGGCATTG |
| ACTB-reverse (rat) | CCTGCTTGCTGATCCACATC |
| SIRT7-forward (rat) | ACGGGCTTGATCTTCAAGGA |
| SIRT7-reverse (rat) | GAGTGAGGTAAGGCAGTGGT |
| GFM2-forward (rat) | AATCCAGGATGTGGCAGTGA |
| GFM2-reverse (rat) | GCAGGCAGTGACCATAGTTG |
| MRPL24-forward (rat) | GGTTGTCCTGGAGGGATTGA |
| MRPL24-reverse (rat) | GATCATGGTCCCTCGGTGAT |
| POLRMT-forward (rat) | CTTCTGGTTGCCACACAACA |
| POLRMT-reverse (rat) | CTCAGCGAACTCCAACAGTG |
| TFB1M-forward (rat) | ACCGAGGGCTTGGAATGTTA |
| TFB1M-reverse (rat) | AGCGTTGGATCAATGTCAGC |
| TFAM-forward (rat) | ATGGACTTCTGCCCACTGAA |
| TFAM-reverse (rat) | GATGCACGCACAGTCTTGAT |
| ACTB-forward (mouse) | GGCTGTATTCCCCTCCATCG |
| ACTB-reverse (mouse) | CCAGTTGGTAACAATGCCATGT |
| SIRT7-forward (mouse) | CAATCCTGTGTTTAGGGTCCAG |
| SIRT7-reverse (mouse) | GTTCACGATGTAGAGTTTGGGTC |
| GFM2-forward (mouse) | TCATTCCGTCATTAACCCTCCT |
| GFM2-reverse (mouse) | AATGCCCCTTTCTCGCTCTTG |
| MRPL24-forward (mouse) | TGAGCCGTCCAGGTTCCATA |
| MRPL24-reverse (mouse) | ACAGAATAGGTGCCAGTCTTCA |
| POLRMT-forward (mouse) | TGGGCGCAAAAGCTAGAGG |
| POLRMT-reverse (mouse) | GTGAAGGGTCCAGAACTCCTG |
| TFB1M-forward (mouse) | CGGGAGATCATTAAGTTGTTCGG |
| TFB1M-reverse (mouse) | GCCCAGGACCCACTTCATAAA |
| TFAM-forward (mouse) | ATTCCGAAGTGTTTTTCCAGCA |
| TFAM-reverse (mouse) | TCTGAAAGTTTTGCATCTGGGT |
| 12.5kb-mito-forward (rat) | AGACGGGTGAGACAGCTGCACCTTTTC |
| 12.5kb-mito-reverse (rat) | CGAGAGCATCAAGTGCAGGCATTAGAG |
| 235bp-mito-forward (rat) | CCTCCCATTCATTATCGCCGCCCTTGC |
| 235bp-mito-reverse (rat) | GTCTGGGTCTCCTAGTAGGTCTGGGAA |
| NAME | SEQUENCE (5’-3’) |
| --- | --- |
| Mitochondrial Mass Quantification | |
| 16S rRNA (mtDNA)-forward (human) | GCCTTCCCCCGTAAATGATA |
| 16S rRNA (mtDNA)-reverse (human) | TTATGCGATTACCGGGCTCT |
| β2-microglobulin (nDNA)-forward (human) | TGCTGTCTCCATGTTTGATGTAT |
| β2-microglobulin (nDNA)-reverse (human) | TCTCTGCTCCCCACCTCTAAGT |
| ND4 (mtDNA)-forward (mouse) | GGAACCAAACTGAACGCCTA |
| ND4 (mtDNA)-reverse (mouse) | ATGAGGGCAATTAGCAGTGG |
| β2-microglobulin (nDNA)-forward (mouse) | TCATTAGGGAGGAGCCAATG |
| β2-microglobulin (nDNA)-reverse (mouse) | ATCCCCTTTCGTTTTTGCTT |
| ND4 (mtDNA)-forward (rat) | CAATCTGCCTACGCCAAACA |
| ND4 (mtDNA)-reverse (rat) | CGGCTGTGAATTCGTTCGTA |
| β2-microglobulin (nDNA)-forward (rat) | TCTGGTGCTTGTCTCTCTGG |
| β2-microglobulin (nDNA)-reverse (rat) | GGGTGGAACTGAGACACGTA |
| Chromatin IP-(qRT)-PCR | |
| SIRT7 promoter region1-forward | TGCCCTGCGCAGTATTTATT |
| SIRT7 promoter region1-reverse | GCTCCCTCTGCTGCTCCT |
| SIRT7 promoter region2-forward | TAGGTCTAGCCAGGGGAGGT |
| SIRT7 promoter region2-reverse | AAGAAGCACCTGCCAGTCTC |
| SIRT7 promoter region3-forward | TGAGACCTCCCTGTTCTTGG |
| SIRT7 promoter region3-reverse | CCAGCCCCTCCTCACTTAG |
| SIRT7 promoter region4-forward | GAGCAGGTCGGGAGAGTAGA |
| SIRT7 promoter region4-reverse | CAGGAGAAGGCTTTCTGTGC |
| SIRT7 promoter region5-forward | ATGGTCAGGTCCCATGTTGT |
| SIRT7 promoter region5-reverse | TGGTACACACCCAGGACACA |
| SIRT7 promoter region6-forward | TTCTGACACAGCGAATCGAG |
| SIRT7 promoter region6-reverse | ACAACATGGGACCTGACCAT |
| SIRT7 promoter region7-forward | GAGTGTTCCTGTCCCCTGTC |
| SIRT7 promoter region7-reverse | CCTGCTGGGTATTCCTCTGA |
| Knock-down | |
| SIRT7 (rat) shRNA target seq-1 | CACCTTTCTGTGAGAACGGAA |
| SIRT7 (rat) shRNA target seq-1 | TAGCCATTTGTCCTTGAGGAA |
| HDAC1 (rat) siRNA-1 | CUCAGGGCACCAAGAGGAATT |
| HDAC1 (rat) siRNA-2 | CGCAGACUCAGGGCACCAATT |
| HDAC2 (rat) siRNA-1 | GCAAGAAGAAAGUGUGCUATT |
| HDAC2 (rat) siRNA-2 | GCAGAUGCAGAGAUUUAAUTT |
| HDAC3 (rat) siRNA-1 | CCAAGAGCCUUAAUGCCUUTT |
| HDAC3 (rat) siRNA-2 | GGAACUUCCCUAUAGUGAATT |
| HDAC4 (rat) siRNA-1 | GCAGUUUCUGGAGAAGCACTT |
| HDAC4 (rat) siRNA-2 | GCAGCAGCACCAGCAGUUUTT |
| HDAC5 (rat) siRNA-1 | GGUUCUACAGAGAGCGAGATT |
| HDAC5 (rat) siRNA-2 | GGACGAGGAGGACUGCAUUTT |
| HDAC6 (rat) siRNA-1 | GCGAAAGAGUAGGCACAAUTT |
| HDAC6 (rat) siRNA-2 | GGGCCAGUCCUCAGAACAATT |
| HDAC7 (rat) siRNA-1 | GCAAGAACUUCGGCAACUUTT |
| HDAC7 (rat) siRNA-2 | GCACACAGCAGACUUUCUATT |
| HDAC8 (rat) siRNA-1 | GCUGGGAGCAGAUACCAUUTT |
| HDAC8 (rat) siRNA-2 | GGACUUGGAUCUACACCAUTT |
| NAME | SEQUENCE (5’-3’) |
| --- | --- |
| Plasmids Construction | |
| SIRT7-forward (Xba I) | GCTCTAGAATGGCAGCCGGGGGTCTGAGCCGCT |
| SIRT7-reverse (EcoR I) | CGGAATTCCGTCACTTTCTTCCTTTTTGTGCGT |
| HDAC1-forward (EcoR I) | CGGAATTCATGGCGCAGACGCAGGGCACCCGGA |
| HDAC1-reverse (BamH I) | CGGGATCCGGCCAACTTGACCTCCTCCTTGACC |
| HDAC2-forward (Xho I) | CCCTCGAGATGGCGTACAGTCAAGGAGGCGGCA |
| HDAC2-reverse (BamH I) | CGGGATCCGGGGTTGCTGAGCTGTTCTGATTTG |
| HDAC3-forward (EcoR I) | CGGAATTCATGGCCAAGACCGTGGCCTATTTCT |
| HDAC3-reverse (BamH I) | CGGGATCCAATCTCCACATCGCTTTCCTTGTCA |
| HDAC4-forward (Xba I) | GCTCTAGAATGAGCTCCCAAAGCCATCCAGATG |
| HDAC4-reverse (EcoR I) | CGGAATTCCAGGGGCGGCTCCTCTTCCATGGGC |
| HDAC5-forward (Xba I) | GCTCTAGAATGAACTCTCCCAACGAGTCGGATG |
| HDAC5-reverse (Hind III) | CCAAGCTTCAGGGCAGGCTCCTGCTCCATGGGC |
| HDAC6-forward (Xba I) | GCTCTAGAATGACCTCAACCGGCCAGGATTCCA |
| HDAC6-reverse (Hind III) | CCAAGCTTGTGTGGGTGGGGCATATCCTCCCCA |
| HDAC7-forward (Xho I) | CCCTCGAGATGGACCTGCGGGTGGGCCAGCGGC |
| HDAC7-reverse (EcoR I) | CGGAATTCGAGATTCATAGGTTCTTCCTCCTCC |
| HDAC8-forward (Xho I) | CCCTCGAGATGGAGGAGCCGGAGGAACCGGCGG |
| HDAC8-reverse (EcoR I) | CGGAATTCGACCACATGCTTCAGATTCCCTTTG |
Supplementary Table 1. Sequences of primers used for siRNA, shRNA, PCR, ChIP, and qPCR analyses used in this study.
